# Rescue of neurologic disease in mucopolysaccharidosis type II mice via AAV-mediated liver delivery of brain-penetrating iduronate-2-sulfatase

**DOI:** 10.1016/j.neurot.2025.e00741

**Published:** 2025-09-18

**Authors:** Xiu Jin, Qin Ye, Xiaoyi Wu, Jing Su, Li Song, Jiamei Fu, Qiuxia Xu, Min Luo, Fanfei Liu, Chengda Ren, Ming Hu, Man Liu, Yifang An, Qiqi Li, Manjun Li, Yang Yang

**Affiliations:** a State Key Laboratory of Biotherapy and Cancer Center, West China Hospital, Sichuan University and Collaborative Innovation Center, Chengdu, Sichuan, China; b Department of Ophthalmology, West China Hospital, Sichuan University, Chengdu, Sichuan, China

**Keywords:** Mucopolysaccharidosis type II, CNS disease, Brain-penetrant IDS, AAV, Gene therapy

## Abstract

Mucopolysaccharidosis type II (MPS II) is a neurodegenerative lysosomal storage disorder (LSD) caused by inherited mutations in the iduronate-2-sulfatase (*IDS*) gene. Approximately two-thirds of patients exhibit severe central nervous system (CNS) involvement and cognitive impairment, which remain unaddressed by conventional enzyme replacement therapy (ERT) due to the inability of wild-type IDS to cross the blood-brain barrier (BBB). To overcome this limitation, we engineered a brain-penetrant IDS variant, eBT-IDS4, which retained enzymatic activity and demonstrated enhanced BBB transcytosis in vitro. We then evaluated a liver-directed gene therapy approach using an adeno-associated virus 8 (AAV8) vector encoding eBT-IDS4 under the control of a liver-specific promoter (LSP) in an adult MPS II mouse model. Intravenous administration of AAV8.LSP.IVS2.eBT-IDS4 resulted in sustained supraphysiological IDS activity and normalization of glycosaminoglycan (GAG) levels in peripheral tissues. Notably, this strategy achieved 89 ​% of wild-type IDS activity in the brain, leading to complete correction of neuropathology and reversal of cognitive deficits in 8-month-old MPS II mice. These findings support a promising, minimally invasive gene therapy strategy for treating MPS II and other neurodegenerative LSDs.

## Graphical abstract

**Figure:**
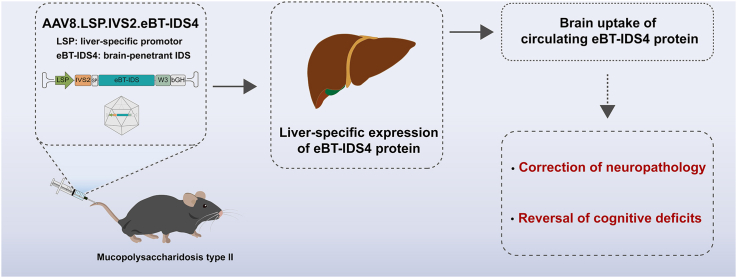

## Introduction

Mucopolysaccharidosis type II (MPS II), also known as Hunter syndrome, is a rare X-linked inherited metabolic disorder [1]. It is caused by mutations in the *IDS* gene, which encodes iduronate-2-sulfatase (IDS), a lysosomal enzyme responsible for the degradation of glycosaminoglycans (GAGs) [2]. Deficiency of IDS leads to progressive accumulation of GAGs throughout the body. MPS II is clinically classified into two forms: a severe form and an attenuated form, both of which present with physical clinical manifestations that include coarsening of facial features, short stature, dysostosis multiplex, joint stiffness, hepatosplenomegaly, and cardiorespiratory symptoms [3]. Approximately two-thirds of patients exhibit the severe form, characterized by significant central nervous system (CNS) involvement and cognitive impairment in addition to somatic manifestations [4].

Enzyme replacement therapy (ERT) has been used to treat MPS II. Although ERT can delay disease progression, it fails to address the neurological manifestations due to the inability of recombinant IDS to cross the blood-brain barrier (BBB) [5,6]. Furthermore, ERT provides insufficient delivery to deep somatic tissues such as the heart, lungs, and bones [[7], [8], [9]], and the formation of neutralizing antibodies may decrease its long-term efficacy or trigger hypersensitivity reactions [10,11]. Recently, several BBB-penetrant IDS fusion strategies have been explored. For example, JR141, an IDS fused to an anti-human transferrin receptor antibody, has been shown to prevent neurodegeneration in preclinical models and clinical trials [12,13]. AGT-182, which fuses IDS with a monoclonal antibody targeting the human insulin receptor, has demonstrated BBB transcytosis in rhesus monkeys [14,15]. A phase I/II clinical trial (NCT02262338) evaluating its safety has been completed, with no results posted yet. DNL310 (NCT04251026), another transferrin receptor-targeted IDS fusion, is currently under clinical investigation. Hematopoietic stem cell transplantation (HSCT), though effective in other forms of MPS, has shown inconsistent outcomes in MPS II and is associated with high morbidity and mortality [[16], [17], [18], [19], [20], [21], [22]]. Thus, there is an urgent need to develop emerging therapies that more effectively target the difficult-to-treat tissues in MPS II, particularly within the central nervous system.

Adeno-associated virus (AAV)-mediated gene therapy has emerged as a promising strategy for MPS II. Clinical trials of RGX-121 (AAV9.CB7.hIDS), which is administered via intracisternal or intracerebroventricular routes (NCT03566043, NCT04571970), aim to evaluate its efficacy against CNS disease manifestations. However, it remains unclear whether delivery via the cerebrospinal fluid (CSF) is sufficient to treat the peripheral and/or CNS manifestations of MPS II. In contrast, liver-directed AAV gene therapy represents an attractive systemic approach in which the liver serves as a biofactory for continuous secretion of therapeutic enzymes into circulation. Indeed, liver-directed AAV gene transfer has demonstrated durable transgene expression and clinical efficacy in inherited metabolic disorders such as hemophilia [23]. In preclinical animal models, AAV-mediated hepatic expression of IDS has led to significant improvements in peripheral tissues but failed to address CNS pathology due to limited BBB penetration [24,25].

To address this challenge, we developed a liver-directed AAV gene therapy that combines AAV-mediated hepatic expression with a brain-penetrant delivery strategy. We first engineered a brain-penetrant IDS variant, eBT-IDS4, which demonstrated efficient BBB transcytosis in vitro and resulted in significantly increased brain IDS activity in 3-month-old MPS II mice. Next, we optimized the regulatory elements (enhancer, intron, and post-transcriptional regulatory elements) of the AAV vector to enhance IDS expression in the liver. Finally, we packaged eBT-IDS4 into an AAV8 vector driven by a liver-specific promoter (LSP) and evaluated its long-term therapeutic efficacy and safety in adult MPS II mice. Systemic administration of AAV8.LSP.IVS2.eBT-IDS4 led to complete correction of both peripheral and CNS manifestations in 8-month-old MPS II mice. These findings highlight a promising, minimally invasive therapeutic strategy with potential to treat both systemic and neurologic disease in MPS II.

## Materials and methods

### Plasmid construction

First, the human IDS sequence was codon optimized and synthesized by GENEWIZ. To generate brain-penetrant variants, a melanotransferrin peptide (DSSHAFTLDELR) [26] was fused to either the C or N-terminus of the codon-optimized IDS sequence via an (EAAAK)_3_ linker, yielding the eBT-IDS1 and eBT-IDS2 sequences. To further enhance brain targeting, two melanotransferrin peptides were fused to the C or N-terminus of the IDS sequence, again using an (EAAAK)_3_ linker; the two melanotransferrin peptides were linked by an XTEN linker (SGSETPGTSESATPES), producing the eBT-IDS3 and eBT-IDS4 variants. Each of these engineered IDS sequences (eBT-IDS1, eBT-IDS2, eBT-IDS3, and eBT-IDS4) was cloned into a plasmid containing a thyroxin-binding globulin (TBG) promoter, a Kozak sequence, and a bovine growth hormone (bGH) polyadenylation signal, all flanked by AAV2 inverted terminal repeats (ITRs), thereby generating the TBG.eBT-IDS1, TBG.eBT-IDS2, TBG.eBT-IDS3, and TBG.eBT-IDS4 constructs. To further increase expression, the intervening sequence 2 (IVS2) intron and the W3 post-transcriptional regulatory sequence were subcloned into the eBT-IDS4 construct, resulting in the TBG.IVS2.eBT-IDS4 construct. Additionally, a liver-specific promoter (LSP) was developed by combining a chimeric enhancer consisting of transthyretin, an alpha micro/bikunin precursor, and apolipoprotein E with the human alpha 1-antitrypsin (hAAT) promoter. Replacing the TBG promoter in the TBG.IVS2.eBT-IDS4 construct with either the hAAT promoter or the LSP produced the hAAT.IVS2.eBT-IDS4 and LSP.IVS2.eBT-IDS4 constructs, respectively. The nucleotide sequences of the melanotransferrin peptide and linkers are listed in Table S1. The sequences of the IDS variants are shown in the Supplemental Sequences.

### Plasmid transfection

HuH7 cells (human hepatoma cell line, JCRB) were maintained in DMEM supplemented with 10 ​% FBS and cultured at 37 ​°C with 5 ​% CO2. The plasmids were transfected into HuH7 cells via the PEIpro DNA Transfection Kit (Polyplus) according to the manufacturer's recommendations. At 48 ​h after transfection, the cell lysates and culture supernatants were harvested for the IDS enzyme activity assay.

### In vitro uptake and transcytosis of enzymes

The cell culture supernatant containing the IDS enzyme was collected from transfected HuH7 cells and quantified via an ELISA kit (R&D System, DY2449-05) according to the manufacturer's instructions. Enzyme uptake and transcytosis assays were performed as previously described [27]. For the uptake assay, human cerebral microvascular endothelial (hCMEC/D3) cells (Millipore) were seeded into well plates at a density of 5 ​× ​10^4^ ​cells/cm^2^ and cultured for 5 days. A standardized amount of IDS enzyme was then added to the hCMEC/D3 monolayer, and IDS activity was measured after 24 ​h of incubation at 37 ​°C. For the transcytosis assay, hCMEC/D3 cells were seeded into collagen I-coated Transwell inserts (Corning, 354236 and 3470) at the same density and cultured for 5 days. After an equal amount of IDS enzyme was added to the Transwell inserts, the plates were incubated at 37 ​°C for 24 ​h, after which IDS activity was measured in both the cells on the Transwell insert and the basolateral medium.

### AAV8 vector production

AAV8 vectors were generated via triple plasmid transfection of human embryonic kidney 293 ​cells (HEK-293, ATCC). All AAV8 vectors were produced as previously described [28]. The genome titer (genome copies (GC)/mL) of the AAV vectors was determined via digital droplet PCR (ddPCR).

### Sex as a biological variable

Male mice were used in this study. Because MPS II is an X-linked genetic disease, affected individuals are almost always males. Only a few female cases with the disorder have been reported [4].

### Systemic vector administration

MPS II knockout mice were purchased from GemPharmatech, and the wild-type mice used in this study were on a C57BL/6J background. All animal protocols were approved by the Experimental Animal Ethics Committee at West China Hospital, Sichuan University, and all procedures were performed in accordance with its guidelines. The mice were maintained on a standard chow diet at the Sichuan University animal facility. Male MPS II mice (4–6 weeks old) were randomly assigned to experimental groups. The AAV vectors were diluted in 200 ​μL of PBS and administered via tail vein injection. Serum was collected via retro-orbital bleeding prior to dosing and then every 1–2 weeks after treatment. Urine samples were obtained by gently pressing the bladder at the time of necropsy. All the mice were euthanized 7 months after injection, at which point blood and tissue samples were collected.

### IDS enzyme activity assay

The tissue and serum samples were immediately stored at −80 ​°C after collection. Serum was used directly for the IDS enzyme activity assay, whereas tissue samples were lysed in buffer (0.9 ​% NaCl, 0.2 ​% Triton X-100, pH 3.5) to obtain protein extracts, which were subsequently quantified via a BCA assay (Thermo Fisher Scientific). IDS activity was determined using a two-step assay with the synthetic substrate 4-methylumbelliferyl α-l-idopyranosiduronic acid 2-sulfate disodium salt (Biosynth Carbosynth, EM03201), as previously described [29]. Briefly, 10 ​μL of sample was incubated with a 1.25 ​mM substrate solution (0.1 ​M sodium acetate, 0.01 ​mM lead acetate, pH 5.0) at 37 ​°C for 4 ​h. Subsequently, 1 ​μg/mL human α-l-iduronidase (R&D Systems, 4119-GH-010) was added, and the reaction was allowed to proceed overnight. The reaction was terminated by adding 200 ​μL of stop buffer (290 ​mM glycine, 180 ​mM citrate, pH 10.9). The fluorescence was measured at an excitation wavelength of 365 ​nm and an emission wavelength of 450 ​nm via a SpectraMax® i3x plate reader (Molecular Devices). Enzyme activity is expressed in nmol per hour per mg protein or mL serum (nmol/h/mg or nmol/h/mL).

### GAG assay

Tissue and urine GAG levels were determined via the Blyscan Glycosaminoglycan Assay Kit (Biocolor) according to the manufacturer's instructions.

### AAV8 biodistribution

DNA was extracted from liver tissue, and total vector genomes were quantified using TaqMan qPCR, as previously described [30].

### Immunohistochemistry

Fresh brain, heart, liver, spleen, lung, and kidney tissues were fixed in 4 ​% paraformaldehyde for 24 ​h, embedded in paraffin, and sectioned at a thickness of 5 ​μm. Sagittal sections of the brain tissue were prepared at 1.56 ​mm lateral from the midline. The sections were used for H&E staining and Alcian blue staining. H&E staining was performed according to the standard protocols, and Alcian blue staining was carried out as described previously [31]. Three representative brain regions, including the cortex, hypothalamus, and brain stem, were selected for Alcian blue staining to assess GAG accumulation. Alcian blue staining was quantified on one brain section per mouse (n ​= ​3 per group) using the built-in color deconvolution algorithm in ImageJ. Results were reported as the ratio of Alcian blue-positive area to total area.

Knee joints were decalcified after fixation in 4 ​% paraformaldehyde for 24 ​h. The decalcified samples were embedded in paraffin and sectioned at 4 ​μm. Sections were stained with 1 ​% toluidine blue aqueous solution for 20 ​min and then examined microscopically.

Fresh brain tissues were fixed in 4 ​% paraformaldehyde, dehydrated, and embedded in Tissue-Tek™ O.C.T. compound (Sakura Finetek, 4583) for snap freezing. 30-μm coronal brain sections (both −1.22 ​mm from bregma) were prepared as follows: after fixation in ice-cold 4 ​% paraformaldehyde for 15 ​min and subsequent washes in PBS, the sections were blocked with a solution of 10 ​% goat serum and 0.5 ​% Triton X-100 for 1 ​h to reduce nonspecific binding. Next, the sections were incubated overnight at 4 ​°C with the appropriate primary antibodies. After being washed with PBS, they were incubated with secondary antibodies conjugated to Alexa Fluor 488 (Abcam, ab150077) or Alexa Fluor 594 (Abcam, ab150160) for 1 ​h at room temperature. Following further washes with PBS, the slides were mounted with DAPI-containing mounting medium and coverslipped. The primary antibodies used were rabbit anti-NeuN (Abcam, ab177487) at a 1:1000 dilution, rat anti-LAMP1 (BD Biosciences, 553792) at a 1:1000 dilution, and rat anti-GFAP (Abcam, ab7260) at a 1:5000 dilution. The secondary antibodies were diluted 1:500. Four representative brain regions, including the motor cortex (M1/M2), striatum, hippocampus (CA3), and hypothalamus, were selected to evaluate GFAP and LAMP1 immunofluorescence. Among these regions, the motor cortex and hypothalamus were subjected to quantitative analysis of GFAP and LAMP1 signals. Quantification was performed using ImageJ software on one brain section per mouse (n ​= ​3 per group). The results were reported as the ratio of the GFAP- or LAMP1-positive area to the total analyzed area.

### Delayed matching-to-place (DMP) dry maze assay

The cognitive performance of MPS II mice was assessed via the DMP dry maze, as previously described [32]. Briefly, the DMP dry maze consists of a circular platform (diameter, 122 ​cm; thickness, 1.2 ​cm) with 40 holes. An escape pipe was secured under one of the holes to allow the mice to escape the platform. Specifically, we defined the area near the escape holes as the target escape zone. Visual cues were attached to each of the four walls for the mouse to use for spatial navigation. To begin the experiment, the mice were placed on the edge of the platform some distance from the escape hole, and an opaque funnel was used to cover the mice. While the opaque funnel was removed, the mice were immediately exposed to bright light (1200 lux) and tonal (2 ​kHz, 85 ​dB) stimuli. In response to these aversive conditions, the mice spontaneously sought out and burrowed into the escape hole. The mice were assessed during four trials per day on 4 consecutive days, with the maximal escape time limited to 3 ​min. Data were collected and analyzed via the ANY-Maze program (SANS).

### Microcomputed tomography (micro-CT)

The zygoma and femur of the mice were scanned via a Quantum GX micro-CT system (PerkinElmer) at 7 months after injection. Images were analyzed via ImageJ.

### Echocardiography

At 7 months post-treatment, high-frequency echocardiography was performed via a Vevo®3100 system (Fujifilm VisualSonics) to evaluate cardiac function. The mice were anesthetized with isoflurane and placed in the supine position on a warming platform, maintaining a heart rate between 400 and 500 beats per minute. M-mode images were acquired from the short-axis view of the left ventricle (LV) to assess its function and dimensions, whereas the aortic arch was visualized via a modified suprasternal view. Parameters, including LV fractional shortening, ejection fraction, heart rate, LV end-systolic diameter, and LV end-diastolic diameter, were analyzed via the Vevo LAB LV analysis tool.

### ALT/AST

Serum ALT and AST levels were measured by an automatic biochemistry analyzer (Cobas 6000 c501, Roche).

### Statistics

GraphPad Prism 10 was used to perform all the statistical tests. The values are expressed as the mean ​± ​SD or mean ​± ​SEM. Statistical analysis was performed via Dunnett's test, as indicated in the Figure legends. In all tests, p values are represented as follows: ∗p value ​< ​0.05; ∗∗p value ​< ​0.01; ∗∗∗p value ​< ​0.001; ∗∗∗∗p value ​< ​0.0001.

## Results

### Development and validation of brain-targeting human IDS enzyme

To enhance the therapeutic efficacy of IDS gene transfer, we developed a codon-optimized version of the human IDS coding sequence (76 ​% nucleotide identity compared with the wild-type coding sequence). Wild-type IDS (IDSwt) or codon-optimized IDS (IDSco) coding sequences (Fig. S1a) were cloned into a hepatocyte-specific expression cassette containing a liver-specific TBG promoter, Kozak sequence, and bGH polyadenylation sequence. These constructs were then transiently transfected into the HuH7 cells. Compared with the IDSwt coding sequence, the IDSco coding sequence showed significantly higher expression and secretion levels, with approximately 8-fold and 5-fold increases in expression and secretion, respectively (Fig. S1b and S1c).

To overcome the limitations of the BBB, we developed two engineered brain-penetrant IDS sequence variants 1 and 2 (eBT-IDS1 and eBT-IDS2) by fusing the melanotransferrin peptide to the C or N-terminus of the IDSco using an (EAAAK)_3_ linker (Fig. S2a). The melanotransferrin peptide has been identified as a brain-targeted peptide that can efficiently cross the BBB and enter the brain parenchyma [26]. Because adding a linker and peptide can alter protein folding and detrimentally affect enzyme activity, the eBT-IDS1 and eBT-IDS2 coding sequences were individually cloned into the same TBG expression cassette and evaluated by transient transfection of HuH7 cells. A construct expressing unmodified IDSco was used as a control. Both eBT-IDS1 and eBT-IDS2 exhibited significantly higher IDS activity in cell lysates and culture supernatants compared to unmodified IDSco, indicating that these modifications did not compromise IDS expression (Fig. S2b and S2c).

To evaluate the in vivo uptake of eBT-IDS1 and eBT-IDS2 by brain tissue, AAV8 vectors encoding either the eBT-IDS1 or eBT-IDS2 transgene under the control of the TBG promoter were packaged and administered to 4- to 6-week-old MPS II mice via intravenous injection (1 ​× ​10^11^ GC/mouse). An AAV8 vector encoding unmodified IDSco was used as a control. Two months post-treatment, IDS proteins were efficiently produced in hepatocytes (Fig. S3a) and secreted into the bloodstream in all treatment groups, with serum IDS activity maintained at supraphysiological levels (Fig. S3b). Although IDS protein was detected in the brain tissues of all treated mice, neither AAV8.TBG.eBT-IDS1 nor AAV8.TBG.eBT-IDS2 resulted in a significant increase in IDS enzyme activity in the brain of MPS II mice compared to AAV8.TBG.IDSco (Fig. S3c). Abnormal accumulation of GAGs is a hallmark of MPS II. GAG levels were normalized in the liver of mice treated with AAV8.TBG.IDSco, AAV8.TBG.eBT-IDS1, and AAV8.TBG.eBT-IDS2, but brain GAG levels were only partially reduced in these treated mice (Fig. S3d and S3e).

**Fig. 1:**
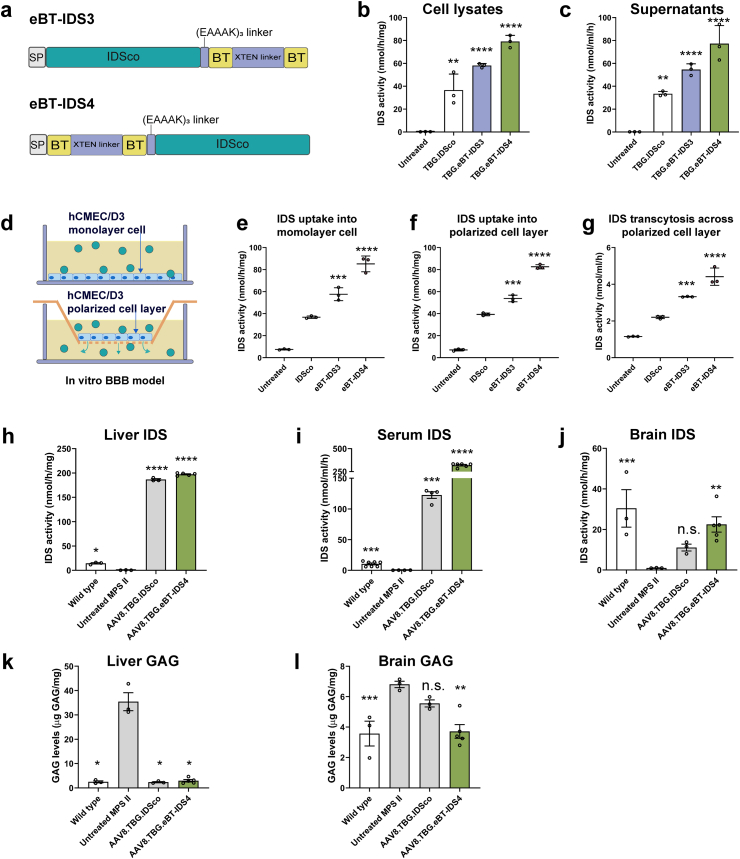
**Development and validation of brain-targeting human IDS enzyme.** (a) Schematic of the eBT-IDS3 or eBT-IDS4 genes. SP, signal peptide; IDSco, codon-optimized IDS; BT, brain-targeted peptide (melanotransferrin peptide). (b and c) IDS enzyme activity in HuH-7 (b) cell lysates and (c) culture supernatants after transfection. The untreated group served as a control. Bars represent mean ​± ​SD, n ​= ​3. Significance was validated with one-way ANOVA, ∗∗p ​< ​0.01, ∗∗∗p ​< ​0.001, ∗∗∗∗p ​< ​0.0001 vs. untreated. (d) Schematic representation of the in vitro enzyme uptake and transcytosis assays. BBB, blood-brain barrier. (e) Uptake of enzymes produced by HuH-7 ​cells added to the growth media of hCMEC/D3 cells grown in monolayer culture for 24 ​h. (f) Uptake of enzymes produced by HuH-7 ​cells added to the growth media of hCMEC/D3 cells grown in Transwell culture for 24 ​h. (g) Transcytosis of enzymes produced by HuH-7 ​cells to the basolateral side of the Transwells. (e–g) Bars represent mean ​± ​SD, n ​= ​3. Significance was validated with one-way ANOVA, ∗∗∗p ​< ​0.001, ∗∗∗∗p ​< ​0.0001 vs. IDSco. (h–j) IDS enzyme activity in (h) liver, (i) serum, and (j) brain at 2 months post-injection. (k and l) GAG levels in the (k) liver and (l) brain at 2 months post-injection. (h–l) Wild-type and untreated mice served as controls. Bars represent mean ​± ​SEM, n ​= ​3–7 mice/group. Significance was validated with one-way ANOVA, n.s. ​= ​not significant, ∗∗p ​< ​0.01, ∗∗∗p ​< ​0.001, ∗∗∗∗p ​< ​0.0001 vs. untreated MPS II.

To further enhance the uptake of IDS enzymes by brain tissue, we developed additional engineered brain-penetrant IDS sequence variants, eBT-IDS3 and eBT-IDS4, by fusing two melanotransferrin peptides to the C or N-terminus of IDS via an (EAAAK)_3_ linker. The two melanotransferrin peptides were linked by a flexible XTEN linker (Fig. 1a). The eBT-IDS3 and eBT-IDS4 sequences were individually cloned into the same TBG expression cassette and evaluated by transient transfection of HuH-7 ​cells. The results showed that eBT-IDS3 and eBT-IDS4 did not compromise IDS enzyme activity. Notably, both variants significantly increased enzyme activity in cell lysates and culture supernatants (Fig. 1b and c). In contrast, fusing two melanotransferrin peptides, either directly in tandem or via the (EAAAK)_3_ rigid linker, significantly reduced IDS enzyme activity (Fig. S4).

Next, we evaluated the uptake of the eBT-IDS3 and eBT-IDS4 proteins by monolayer-cultured hCMEC/D3 cells, a type of brain capillary endothelial cell (Fig. 1d). Relative enzyme levels were quantified via ELISA. In this monolayer culture, the uptake of eBT-IDS3 and eBT-IDS4 increased by 1.6-fold and 2.3-fold, respectively, compared with that of unmodified IDSco (Fig. 1e). We subsequently established an in vitro BBB model by culturing hCMEC/D3 cells on Transwell inserts (Fig. 1d). We evaluated both the uptake and transcytosis of eBT-IDS3 and eBT-IDS4 proteins across the polarized cell layer. In the BBB model, the uptake of eBT-IDS3 and eBT-IDS4 increased by 1.4-fold and 2.1-fold, respectively, compared with that of unmodified IDSco, similar to when the cells were cultured in monolayers (Fig. 1f). Additionally, transcytosis to the basolateral compartment increased by 1.5-fold for eBT-IDS3 and 2-fold for eBT-IDS4 (Fig. 1g). These results indicate that the eBT-IDS4 protein remains enzymatically active and efficiently crosses the BBB in vitro. On the basis of these findings, the eBT-IDS4 coding sequence was selected for subsequent studies.

To evaluate the in vivo uptake of eBT-IDS4 by brain tissue, an AAV8 vector encoding the eBT-IDS4 transgene driven by the TBG promoter was packaged and administered to 4- to 6-week-old MPS II mice via intravenous injection (1 ​× ​10^11^ GC/mouse). An AAV8 vector encoding unmodified IDSco was used as a control. Two weeks post-injection, supraphysiological serum IDS enzyme activity was observed in both the AAV8.TBG.IDSco and AAV8.TBG.eBT-IDS4 treatment groups (Fig. S5a). Notably, a significant increase in brain IDS enzyme activity was detected only in the AAV8.TBG.eBT-IDS4 group (Fig. S5b). We further assessed the therapeutic efficacy of AAV8.TBG.eBT-IDS4 at two months post-treatment. The results showed that the liver-expressed eBT-IDS4 enzymes were both active and efficiently secreted into the bloodstream (Fig. 1h and i). Serum IDS activity in the AAV8.TBG.eBT-IDS4-treated group reached nearly 30-fold higher than wild-type levels (Fig. 1i), and brain IDS enzyme activity reached 73 ​% of the wild-type level (Fig. 1j). Moreover, GAG levels in both liver and brain tissues were normalized in the AAV8.TBG.eBT-IDS4-treated group (Fig. 1k and l). These results suggest that eBT-IDS4 can be efficiently taken up by brain tissue, which leads to a reduction in brain GAG levels in 3-month-old MPS II mice.

### Enhancing IDS expression through vector design

**Fig. 2:**
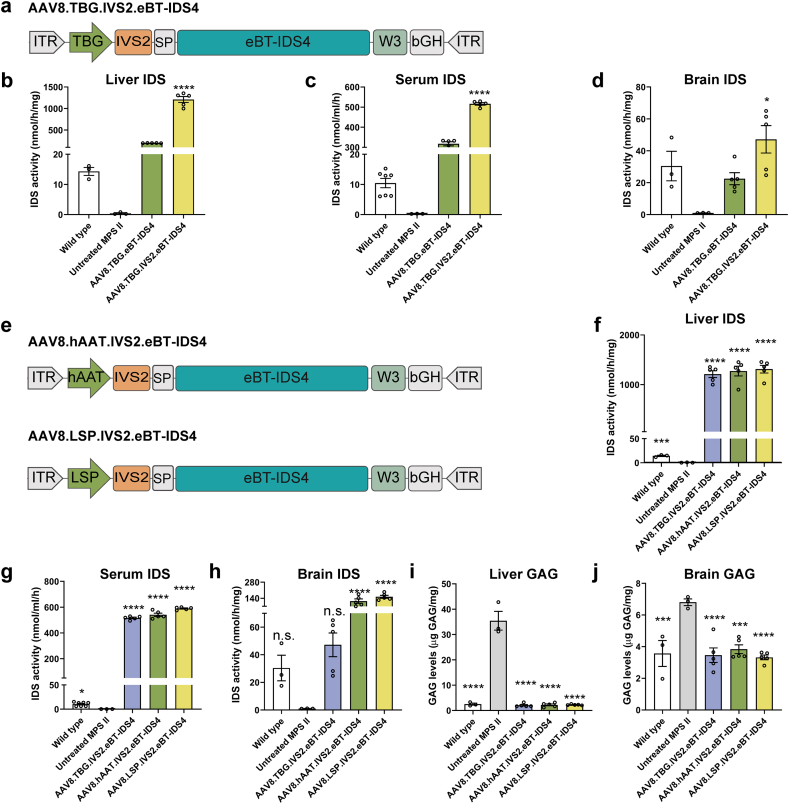
**Enhancing IDS expression through AAV vector design.** (a) Schematic of AAV8 vector genomes encoding the eBT-IDS4 transgene driven by the TBG promoter, with the addition of IVS2 and W3 regulatory elements. ITR, inverted terminal repeats; SP, signal peptide; bGH, polyadenylation signal. (b–d) IDS enzyme activity in the (b) liver, (c) serum, and (d) brain after 2 months of treatment. Wild-type and untreated MPS II mice served as controls. Bars represent mean ​± ​SEM, n ​= ​5 mice/group. Significance was validated with one-way ANOVA, ∗p ​< ​0.01, ∗∗∗∗p ​< ​0.0001 vs. AAV8.TBG.eBT-IDS4. (e) Schematic of AAV8 vector genomes encoding the eBT-IDS4 transgene, driven by the hAAT promoter or liver-specific promoter (LSP), with the addition of IVS2 and W3 regulatory elements. (f–h) IDS enzyme activity in the (f) liver, (g) serum, and (h) brain after 2 months of treatment. (i and j) GAG levels in the (i) liver and (j) brain at 2 months post-injection. (f–j) Wild-type and untreated MPS II mice served as controls. Bars represent mean ​± ​SEM, n ​= ​3–7 mice/group. Significance was validated with one-way ANOVA, n.s. ​= ​not significant, ∗∗∗p ​< ​0.001, ∗∗∗∗p ​< ​0.0001 vs. untreated MPS II.

To enhance IDS expression mediated by AAV vectors, we assessed the in vivo effects of adding two regulatory elements: the intron sequence IVS2 of the human beta-globin gene and W3 (a truncated woodchuck hepatitis virus post-transcriptional regulatory element). Both of these elements have been previously reported to increase AAV vector-mediated transgene expression [33,34]. We produced an AAV8 vector encoding the eBT-IDS4 transgene with IVS2 and W3 (Fig. 2a). The AAV8 vector was intravenously administered to 4- to 6-week-old MPS II mice at a dose of 1 ​× ​10^11^ GC/mouse, with an AAV8 vector lacking these elements serving as a control. Two months post-injection, mice treated with the AAV8 vector containing IVS2 and W3 exhibited a significant increase in liver IDS enzyme activity (Fig. 2b), with a 1.6-fold increase in serum IDS activity and a 2.1-fold increase in brain IDS activity compared with the vector lacking IVS2 and W3 (Fig. 2c and d).

Next, we optimized the promoter by developing a liver-specific promoter (LSP) that combines a chimeric enhancer consisting of transthyretin, an alpha micro/bikunin precursor, and apolipoprotein E with the hAAT promoter. To evaluate the effect of LSP on IDS expression in vivo, we produced AAV8 vectors encoding the eBT-IDS4 transgene driven by TBG, hAAT, or LSP (Fig. 2e). These AAV8 vectors were injected intravenously (1 ​× ​10^11^ GC/mouse) into 4- to 6-week-old MPS II mice. Two months post-injection, the AAV8.LSP.IVS2.eBT-IDS4-treated group showed robust hepatic expression (Fig. 2f) and efficient secretion of IDS enzymes, with serum IDS activity reaching 56-fold of wild-type levels (Fig. 2g). In contrast, the serum IDS enzyme activity in the AAV8.TBG.IVS2.eBT-IDS4- and AAV8.hAAT.IVS2.eBT-IDS4-treated groups reached 49- and 51-fold of the wild-type levels, respectively (Fig. 2g). Notably, brain IDS activity in the AAV8.LSP.IVS2.eBT-IDS4 group was supraphysiological, exhibiting 3.3-fold and 1.3-fold increases compared with those in the TBG and hAAT groups, respectively (Fig. 2h). GAG levels were normalized in the liver and brain of mice treated with AAV8.TBG.IVS2.eBT-IDS4, AAV8.hAAT.IVS2.eBT-IDS4 or AAV8.LSP.IVS2.eBT-IDS4 (Fig. 2i and j). Overall, through IDS sequence engineering and vector design, we developed an AAV8 vector encoding eBT-IDS4 transgene driven by an engineered LSP. Intravenous administration of this vector efficiently restored brain IDS activity, leading to normalization of GAG levels in the brains of 3-month-old MPS II mice.

### Liver-directed AAV8.LSP.IVS2.eBT-IDS4 gene therapy significantly restores IDS enzyme activity in the brain and peripheral tissues

**Fig. 3:**
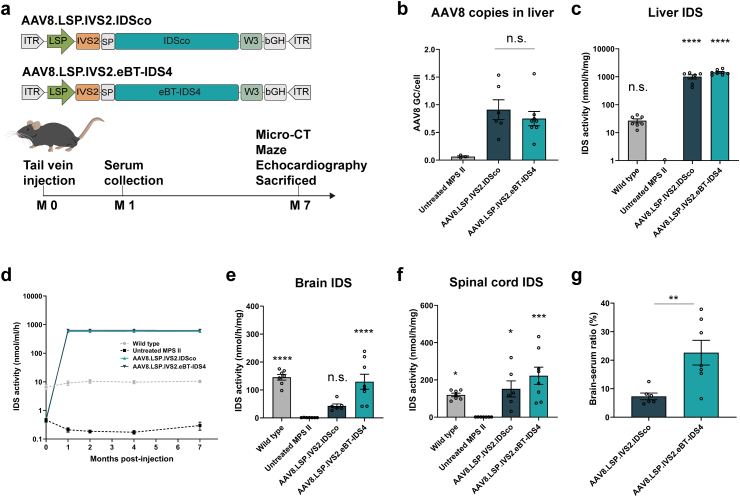
**AAV8.LSP.IVS2.eBT-IDS4 significantly restores IDS enzyme activity in the brain and peripheral tissues of 8-month-old MPS II mice.** (a) Schematic of AAV8.LSP.IVS2.IDSco and AAV8.LSP.IVS2.eBT-IDS4 vector genomes for tail vein delivery (upper panel) and timeline of the experimental setup (lower panel). IDSco, codon-optimized IDS; eBT-IDS4, brain-penetrant IDS sequence variants 4; IVS2 and W3, regulatory elements; ITR, inverted terminal repeats; SP, signal peptide; bGH, polyadenylation signal. (b) Vector genome copy number in the liver at 7 months post-injection, n ​= ​3–8 mice/group. Significance was validated with one-way ANOVA, n.s. ​= ​not significant. (c–f) IDS enzyme activity in tissues at 7 months post-treatment, including (c) liver, (d) serum, (e) brain, and (f) spinal cord. Significance was validated with one-way ANOVA, n.s. ​= ​not significant, ∗p ​< ​0.05, ∗∗∗p ​< ​0.001, ∗∗∗∗p ​< ​0.0001 vs. untreated MPS II. (g) IDS enzyme activity in the brain relative to serum, expressed as a percentage, at 7 months post-injection. Significance was validated with a two-tailed *t*-test, ∗∗p ​< ​0.01. (c–g) Wild-type and untreated mice served as controls. Bars represent mean ​± ​SEM, n ​= ​6–8 mice/group.

To evaluate the long-term therapeutic efficacy of liver-directed gene therapy in MPS II mice, we produced AAV8 vectors encoding either the eBT-IDS4 or unmodified IDSco transgenes driven by the LSP (AAV8.LSP.IVS2.eBT-IDS4 and AAV8.LSP.IVS2.IDSco). These vectors were intravenously injected (1 ​× ​10^11^ GC/mouse) into 4- to 6-week-old MPS II mice. After undergoing micro-CT, echocardiographic, and behavioral analyses, the mice were sacrificed at 7 months post-treatment for biochemical analysis of the brain and peripheral organs (Fig. 3a). Viral copy numbers were detected in the livers of treated mice, with no significant difference between AA8V.LSP.IVS2.eBT-IDS4 and AAV8.LSP.IVS2.IDSco groups (Fig. 3b). Supraphysiological levels of IDS activity were observed in both the liver and serum (Fig. 3c and d), with mean serum activities of 627.8 ​± ​9.2 ​nmol/h/mL and 572.8 ​± ​42.3 ​nmol/h/mL for AAV8.LSP.IVS2.eBT-IDS4 and AAV8.LSP.IVS2.IDSco groups, respectively, at 7 months post-treatment (Fig. 3d). These results indicated that liver-expressed eBT-IDS4 and IDSco enzymes were active and continuously secreted into the bloodstream.

To evaluate the uptake of liver-secreted eBT-IDS4 and IDSco enzymes by the CNS at 7 months post-treatment, we quantified enzyme activity in the brain and spinal cord. Compared to untreated MPS II mice, brain IDS activity was significantly increased in treated mice, reaching 89 ​% and 29 ​% of wild-type levels in the AAV8.LSP.IVS2.eBT-IDS4 and AAV8.LSP.IVS2.IDSco groups, respectively (Fig. 3e). IDS activity in the spinal cords of all treated mice was restored to wild-type levels (Fig. 3f). In addition, supraphysiological IDS activity was detected in the choroid plexus at 8 months post-treatment (Fig. S6a). The efficiency of IDS transport from the circulation into the brain parenchyma was assessed at 7 months post-treatment, revealing a significant difference between the AAV8.LSP.IVS2.eBT-IDS4 group (22.6 ​%) and the AAV8.LSP.IVS2.IDSco group (7.3 ​%) (Fig. 3g). All treated mice exhibited supraphysiological IDS activity in peripheral tissues (heart, spleen, lungs, and kidneys). Specifically, enzyme activity in the AAV8.LSP.IVS2.eBT-IDS4 group was 68-fold (heart), 14-fold (spleen), 5-fold (lungs), and 11-fold (kidneys) higher than those of wild-type mice, whereas the AAV8.LSP.IVS2.IDSco group reached 46-fold (heart), 14-fold (spleen), 5-fold (lungs), and 8-fold (kidneys) increases (Fig. S6b–S6e). Overall, these findings demonstrate that high and consistent levels of the IDS enzyme secreted into the bloodstream are effectively taken up by both the brain and peripheral tissues.

### Liver-directed AAV8.LSP.IVS2.eBT-IDS4 gene therapy normalizes GAG accumulation in the brain and peripheral tissues

**Fig. 4:**
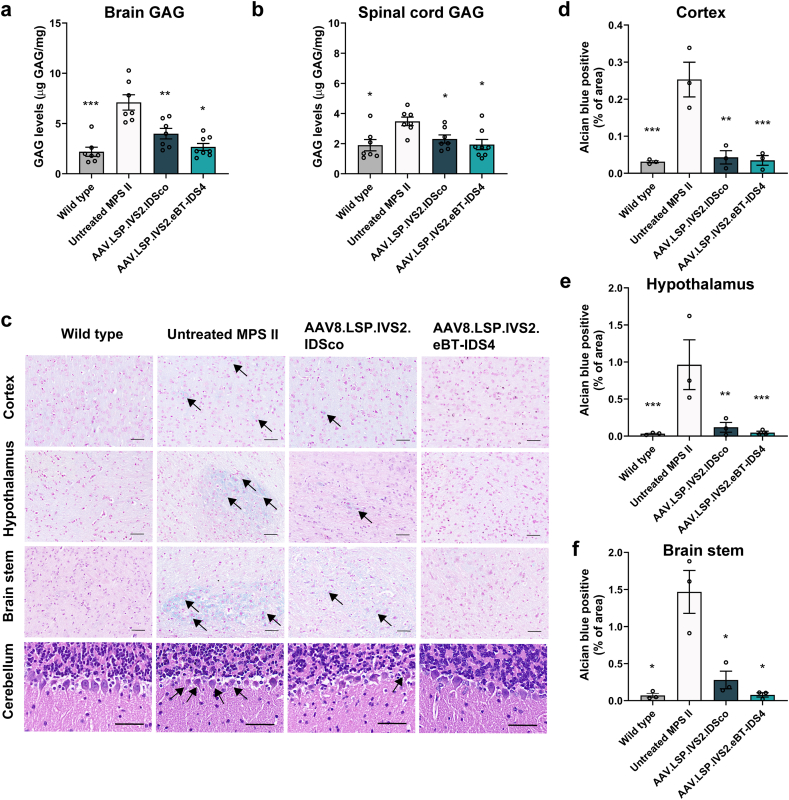
**AAV8.LSP.IVS2.eBT-IDS4 normalizes GAG levels in the brains of 8-month-old MPS II mice.** (a and b) GAG levels in the (a) brain and (b) spinal cord at 7 months post-treatment. Bars represent mean ​± ​SEM, n ​= ​6–8 mice/group. Significance was validated with one-way ANOVA, ∗p ​< ​0.05, ∗∗p ​< ​0.01, ∗∗∗p ​< ​0.001 vs. untreated MPS II. (c) Alcian blue staining of the cortex, hypothalamus, and brain stem, and H&E staining of the cerebellum at 7 months post-treatment. Scale bars: 50 ​μm. Black arrows indicate vacuolation of Purkinje neurons or GAG stained blue. (d–f) Quantification of Alcian blue staining in the (d) cortex, (e) hypothalamus, and (f) brain stem. Data was reported as Alcian blue-positive area/total area. Bars represent mean ​± ​SEM, n ​= ​3 mice/group. Significance was validated with one-way ANOVA, ∗p ​< ​0.05, ∗∗p ​< ​0.01, ∗∗∗p ​< ​0.001 vs. untreated MPS II.

To determine whether the uptake of IDS enzymes by the brain and peripheral tissues at 7 months post-treatment could improve GAG accumulation, we quantified GAG levels in the CNS (brain and spinal cord) and peripheral tissues (heart, liver, spleen, lungs, and kidneys) at 7 months post-treatment. In 8-month-old untreated MPS II mice, GAG levels were significantly increased in both the CNS and peripheral tissues compared with those in wild-type mice (Fig. 4a and b and Fig. S7a–S7e). AAV8.LSP.IVS2.eBT-IDS4 normalized brain GAG levels, whereas AAV8.LSP.IVS2.IDSco reduced brain GAG levels by only 63.4 ​% (Fig. 4a). Moreover, all the treated groups exhibited normalized GAG accumulation in the spinal cord and peripheral tissues (Fig. 4b and Fig. S7a–S7e). These results were consistent with the normalization of urinary GAGs in AAV8.LSP.IVS2.eBT-IDS4-treated mice and the near normalization of urinary GAGs in AAV8.LSP.IVS2.IDSco-treated mice (Fig. S7f).

We further evaluated the tissue GAG content via Alcian blue staining of the brain, heart, liver, spleen, lung, and kidney at necropsy. Consistent with the biochemical results, the GAG content was significantly higher in most tissues of untreated MPS II mice than in those of wild-type control mice (Fig. 4c–f and Fig. S8). In the brain, AAV8.LSP.IVS2.eBT-IDS4 restored the GAG content to wild-type levels, whereas AAV8.LSP.IVS2.IDSco significantly improved but did not fully normalize the brain GAG content (Fig. 4c–f). In addition, normalization of the GAG content was observed in the heart, liver, spleen, lung, and kidney of all treated mice (Fig. S8).

GAG accumulation leads to the formation of characteristic vacuolation in macrophages or neurons. To examine these morphological abnormalities, we performed H&E staining on tissues at necropsy. In 8-month-old untreated II mice, significant vacuolization was observed in Purkinje cells in the brain and macrophages in peripheral tissues (heart, liver, spleen, lung, and kidney) (Fig. 4c and Fig. S9). In the brain, AAV8.LSP.IVS2.eBT-IDS4 completely prevented Purkinje cell vacuolation, whereas a few vacuolated Purkinje cells persisted in the mice treated with AAV8.LSP.IVS2.IDSco (Fig. 4c). However, both treatments corrected vacuolation in peripheral tissues (Fig. S9). Chondrocyte vacuolization was assessed by toluidine blue staining. In the growth plate of femurs from 8-month-old MPS II mice, we observed GAG storage vesicles along with a disorganized, ballooned, and vacuolated columnar structure. In contrast, in mice treated with either AAV8.LSP.IVS2.eBT-IDS4 or AAV8.LSP.IVS2.IDSco, chondrocyte vacuolization was reduced, and the column structure of the growth plate was improved (Fig. S10).

### Liver-directed AAV8.LSP.IVS2.eBT-IDS4 gene therapy improves lysosomal swelling and astrogliosis in the brain

**Fig. 5:**
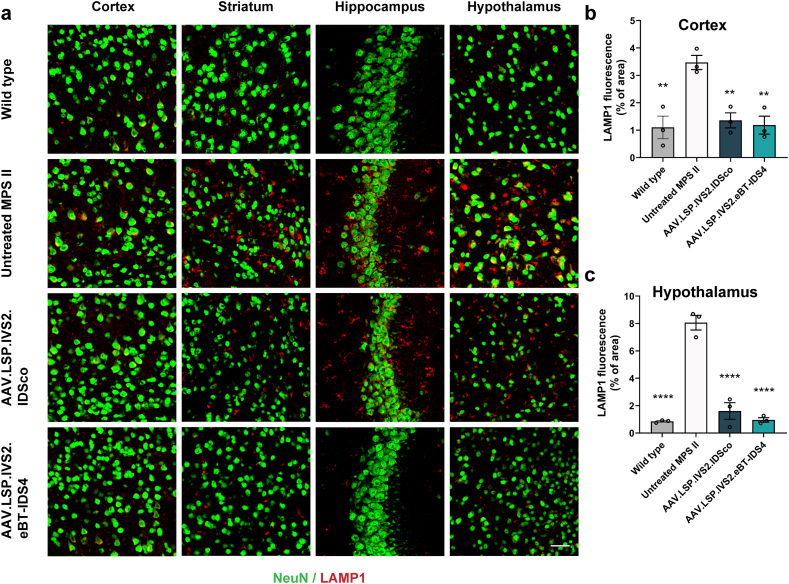
**AAV8.LSP.IVS2.eBT-IDS4 corrects lysosomal compartment size in the brains of 8-month-old MPS II mice.** (a) Representative images of brain sections from the motor cortex (M1/M2), striatum, hippocampus (CA3), and hypothalamus of control and treated mice stained with NeuN (green) and LAMP1 (red). Scale bars: 50 ​μm. (b and c) Quantification of LAMP1 immunofluorescence in the (b) cortex and (c) hypothalamus. Data was reported as LAMP1 positive area/total area. Bars represent mean ​± ​SEM, n ​= ​3 mice/group. Significance was validated with one-way ANOVA, ∗∗p ​< ​0.01, ∗∗∗∗p ​< ​0.0001 vs. untreated MPS II.

To assess the therapeutic effect of AAV8.LSP.IVS2.eBT-IDS4 and AAV8.LSP.IVS2.IDSco on lysosomal swelling in the brain, we performed immunohistochemical staining using the lysosomal marker LAMP1 (lysosomal-associated membrane protein 1, red) and the neuronal marker NeuN (green). Compared with wild-type mice, 8-month-old MPS II mice showed strong colocalization of NeuN and LAMP1 in the cortex, striatum, hippocampus, and hypothalamus, with increased LAMP1 levels indicating a significant lysosomal burden in neurons (Fig. 5a–c). AAV8.LSP.IVS2.IDSco only partially reduced lysosomal substrate accumulation in these regions, which correlated strongly with the levels of GAGs detected in the brain. AAV8.LSP.IVS2.eBT-IDS4 normalized lysosomal swelling in neurons of the cortex, striatum, hippocampus, and hypothalamus (Fig. 5a–c).

**Fig. 6:**
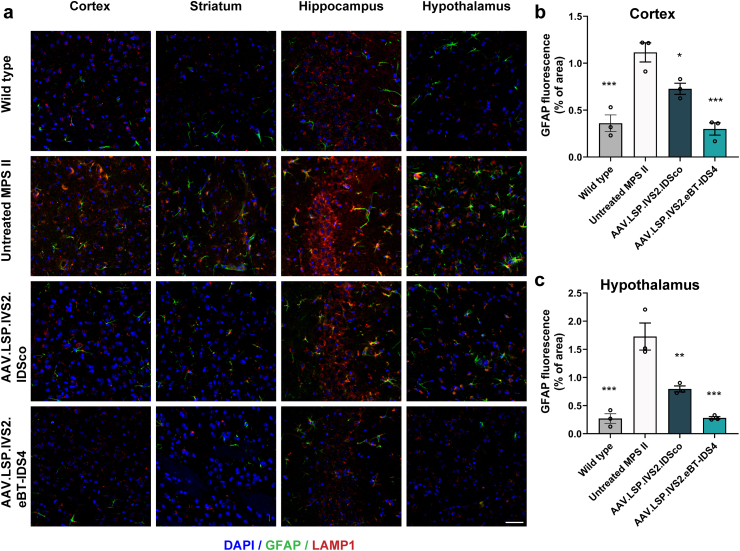
**AAV8.LSP.IVS2.eBT-IDS4 corrects astrogliosis in the brains of 8-month-old MPS II mice.** (a) Representative images of brain sections from the motor cortex (M1/M2), striatum, hippocampus (CA3), and hypothalamus of control and treated mice stained with NeuN (green) and LAMP1 (red). Scale bars: 50 ​μm. (b and c) Quantification of LAMP1 immunofluorescence in the (b) cortex and (c) hypothalamus. Data was reported as GFAP positive area/total area. Bars represent mean ​± ​SEM, n ​= ​3 mice/group. Significance was validated with one-way ANOVA, ∗p ​< ​0.05, ∗∗p ​< ​0.01, ∗∗∗p ​< ​0.001 vs. untreated MPS II.

Astrocytes have been found to mediate a strong neuroinflammatory response in MPS disorders [35]. We therefore performed immunohistochemical staining using the astrocytic markers GFAP (glial fibrillary-associated protein; green) and LAMP1 (red). Compared with those in wild-type mice, extensive astrogliosis was evident in 8-month-old MPS II mice, with significantly increased GFAP staining in the cortex, striatum, hippocampus, and hypothalamus (Fig. 6a–c). Moreover, strong colocalization of GFAP and LAMP1 was observed, indicating that alongside neurons, astrocytes also exhibit significant lysosomal swelling. Notably, AAV8.LSP.IVS2.eBT-IDS4 eliminated astrogliosis and normalized LAMP1 staining to wild-type levels in these regions, whereas AAV8.LSP.IVS2.IDSco only partially reduced the number of reactive astrocytes (Fig. 6a–c).

### Liver-directed AAV8.LSP.IVS2.eBT-IDS4 gene therapy improves neurobehavioral deficits

**Fig. 7:**
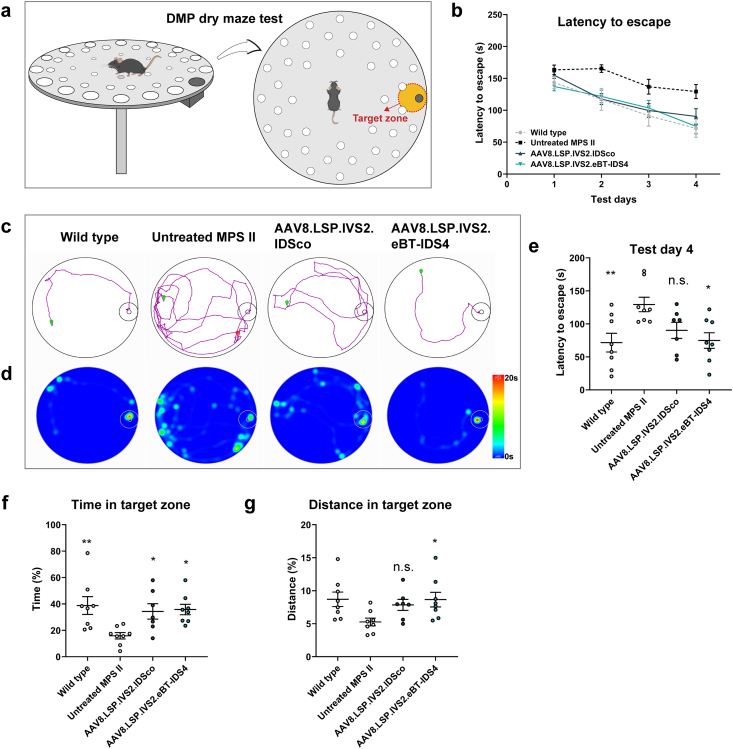
**AAV8.LSP.IVS2.eBT-IDS4 corrects the cognitive deficits of 8-month-old MPS II mice.** (a) Schematic of the delayed matching-to-place (DMP) dry maze assay. The unique escape hole is shown in black, and the orange zone indicates the defined target escape zone. (b) Escape time of the mice from day 1 to day 4 of the DMP dry maze test. (c) Representative track plot on day 4 of the DMP dry maze test. The starting point is shown in green, and the ending point is shown in red. The endpoint is not displayed if the mouse enters the hole. (d) Heatmap corresponding to the representative track plot on day 4 of the test. (e) Escape time on day 4 of the DMP dry maze test. (f and g) Percentages of (f) time and (g) distance spent exploring the target escape zone. (b, e-g) Wild-type and untreated mice served as controls. Bars represent mean ​± ​SEM; n ​= ​7–8 mice/group. Significance was validated with one-way ANOVA, n.s. ​= ​not significant; ∗p ​< ​0.05, ∗∗p ​< ​0.01, ∗∗∗p ​< ​0.001 vs. untreated MPS II.

To evaluate the efficacy of the AAV8.LSP.IVS2.eBT-IDS4 and AAV8.LSP.IVS2.IDSco on cognitive deficits, we performed the DMP dry maze test, which assesses spatial learning and memory abilities and has been extensively used in mouse models of Alzheimer's disease and MPS I [31,36,37]. In this test, mice are challenged to find an escape route on a high platform (Fig. 7a). The study was performed at 7 months post-treatment. Compared with age-matched wild-type controls, untreated MPS II mice exhibited significant cognitive impairments after 4 days of training, as evidenced by longer escape search times and a significantly reduced percentage of time and distance spent in the target escape zone (Fig. 7b–g). Both AAV8.LSP.IVS2.eBT-IDS4 and AAV8.LSP.IVS2.IDSco treatments had a positive effect on cognitive symptoms in the DMP dry maze associated with MPS II (Fig. 7b). On test day 4, the spatial learning and memory performance of the AAV8.LSP.IVS2.eBT-IDS4-treated mice were comparable to that of the wild-type mice (Fig. 7c–g). Compared with untreated MPS II mice, AAV8.LSP.IVS2.eBT-IDS4-treated mice demonstrated significant improvements in the mean escape latency as well as in the percentage of time and distance traversing the target escape zone (Fig. 7c–g). Although AAV8.LSP.IVS2.IDSco also significantly improved cognitive performance, but it was not as effective as the AAV8.LSP.IVS2.eBT-IDS4 (Fig. 7c–g). These results indicate a phenotypic rescue of cognitive symptoms in AAV8.LSP.IVS2.eBT-IDS4-treated mice.

### Liver-directed AAV8.LSP.IVS2.eBT-IDS4 and AAV8.LSP.IVS2.IDSco gene therapy corrects bone and heart disease

**Fig. 8:**
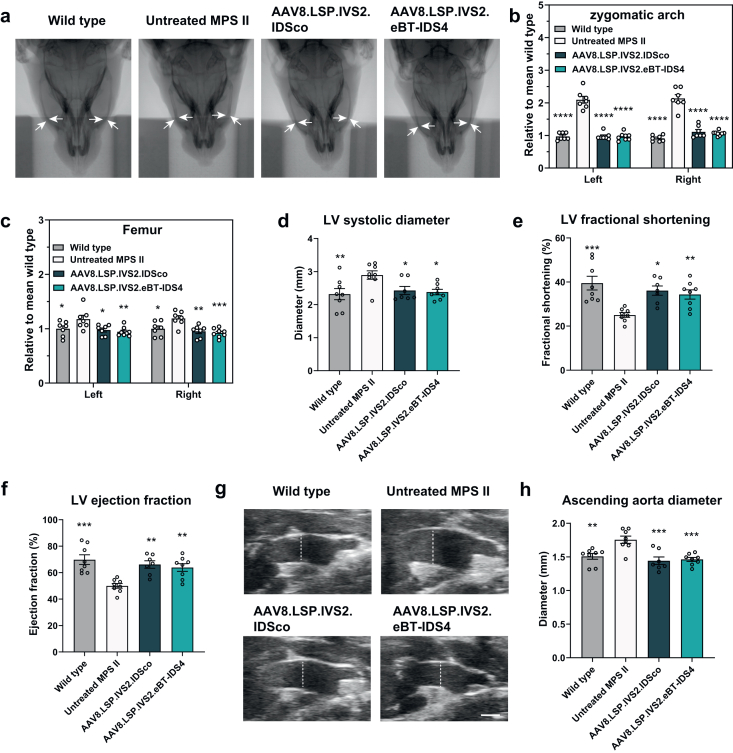
**AAV8.LSP.IVS2.eBT-IDS4 and AAV8.LSP.IVS2.IDSco corrects bone and heart disease in 8-month-old MPS II mice.** (a) Micro-CT images showing a dilated zygomatic arch in 8-month-old MPS II mice (white arrows). (b and c) Quantification of (b) zygomatic arch width and (c) femur width. (d–f) Measurements of the left ventricular (LV) (d) systolic diameter, (e) fractional shortening, and (f) ejection fraction in 8-month-old MPS II mice. (g) Representative ultrasound images of the ascending aortas of 8-month-old MPS II mice. (h) Quantification of the ascending aorta diameter. (b-f, h) Wild-type and untreated mice served as controls. Bars represent mean ​± ​SEM, n ​= ​7–8 mice/group. Significance was validated with one-way ANOVA, n.s. ​= ​not significant; ∗p ​< ​0.05, ∗∗p ​< ​0.01, ∗∗∗p ​< ​0.001, ∗∗∗∗p ​< ​0.0001 vs. untreated MPS II.

All MPS II patients suffer from severe somatic symptoms, including skeletal abnormalities and cardiorespiratory symptoms [3,38]. Micro-CT revealed that 8-month-old untreated MPS II mice exhibited widened zygomatic arches and femurs, approximately 2.3- and 1.2-fold wider, respectively, compared with age-matched wild-type mice (Fig. 8a–c). Treatment with either AAV8.LSP.IVS2.eBT-IDS4 or AAV8.LSP.IVS2.IDSco normalized the enlarged zygomatic arch and femur widths, suggesting significant skeletal rescue (Fig. 8a–c).

Cardiovascular disease in MPS II patients often manifests as heart valve thickening, impaired cardiac function, and aortic dilatation [39]. Echocardiographic analysis revealed that the LV ejection fraction and shortening fraction, which were significantly reduced in untreated MPS II mice, were restored to normal in all the treated groups (Fig. 8d–f). Furthermore, the diameter of the ascending aorta in the treated mice was significantly reduced compared with that in the untreated MPS II group (Fig. 8g and h). These data indicate that liver-directed gene therapy with AAV8.LSP.IVS2.eBT-IDS4 and AAV8.LSP.IVS2.IDSco can completely correct cardiac function and prevent aortic dilatation in MPS II mice.

### Mice treated with liver-directed gene therapy do not yield an immune response to human IDS, and no hepatotoxicity is observed

To determine whether liver-directed gene therapy induces tolerance to human IDS post-treatment, we analyzed serum from mice that received either AAV8.LSP.IVS2.eBT-IDS4 or AAV8.LSP.IVS2.IDSco. Compared with untreated MPS II mice, no significant immune response to these enzymes was observed throughout the treatment period (Fig. S11a and S11b). Finally, hepatotoxicity was assessed in treated mice. Seven months after injection, the serum alanine aminotransferase (ALT) and aspartate aminotransferase (AST) levels remained within normal ranges (Fig. S11c and S11d), with no indications of hepatocellular injury or hepatocellular carcinoma (HCC) (Fig. S11e). Although no side effects were observed in our study, the number of animals analyzed was limited. Therefore, further studies with larger cohorts are warranted to better assess in vivo toxicity.

## Discussion

Currently, finding a suitable therapeutic option to address the neurological manifestations affecting approximately two-thirds of MPS II patients remains a significant challenge. Previous gene therapy studies that combined enzyme engineering to enable BBB penetration have demonstrated significant improvement in CNS manifestations in various MPS mouse models. For instance, lentiviral-mediated hematopoietic stem cell gene therapy delivering BBB-penetrant enzymes has been shown to correct neurological manifestations in MPS I or MPS II mice [27,40,41]. Similarly, liver-directed AAV delivery of BBB-penetrant enzymes has also been demonstrated to rescue brain lesions in MPS I or MPS IIIA mice [31,42]. In this study, we developed a brain-penetrant IDS variant (eBT-IDS4) that was not compromised in expression, secretion, or enzyme activity and exhibited a 2-fold increase in in vitro BBB transcytosis compared with unmodified IDSco (Fig. 1g). To further enhance therapeutic efficacy, we optimized the regulatory elements (enhancer, intron, and post-transcriptional regulatory elements) of the AAV vector, resulting in increased hepatic expression of eBT-IDS4. Importantly, 7 months after injection, treatment with AAV8.LSP.IVS2.eBT-IDS4 restored brain IDS activity in MPS II mice to 89 ​% of wild-type levels. This resulted in complete correction of brain GAG accumulation, neuroinflammation, cognitive deficits, and other somatic manifestations associated with MPS II. Notably, AAV8.LSP.IVS2.eBT-IDS4 achieved superior efficacy compared with AAV8.LSP.IVS2.IDSco, which resulted in only partial neurological improvement. Thus, AAV8.LSP.IVS2.eBT-IDS4 provides a potential therapy for MPS II patients.

Although AAV is considered non-pathogenic, high systemic doses can cause hepatotoxicity [43,44]. In a recent AAV8 gene therapy trial for X-linked myotubular myopathy (NCT03199469), three patients in the high-dose group (3 ​× ​10^14^ vg/kg) died from progressive liver failure, leading to the termination of the trial. Similarly, acute liver failure and death occurred in spinal muscular atrophy patients treated with Zolgensma® [45]. Therefore, dose reduction has become a major focus in current AAV-based gene therapy. In this study, a dose of 1 ​× ​10^11^ vg per mouse (approximately 5 ​× ​10^12^ vg/kg) was used, which falls within the safety range and is less than one-tenth of the dose used in commercial AAV gene therapies and was found to be sufficient for the MPS II model mice.

Studies have shown that the enhancer and promoter design used in recombinant AAV vectors is correlated with HCC risk [46]. High-risk promoters such as chicken beta-actin globin (CAG) and TBG have been reported to be associated with tumor formation [[47], [48], [49], [50]], whereas the hAAT promoter exhibits no oncogenicity, even following integration at the Rian locus [51,52]. In our study, we designed a liver-specific promoter (LSP) that achieved higher transgene expression efficiency compared with TBG and hAAT, with no evidence of hepatotoxicity or HCC in treated mice (Fig. S11). In addition, foreign proteins and enzymes (e.g., ERT) can trigger the release of inhibitory antibodies, thereby reducing therapeutic efficacy [10]. Here, IgG antibodies against human IDS were undetectable following liver-directed AAV gene therapy, suggesting that our approach may reduce the risk of adverse effects.

As previously reported, wild-type mice presented higher IDS levels in brain tissue than in other analyzed tissues [27,29,53,54]. Achieving wild-type IDS levels in the murine brain remains challenging regardless of the gene therapy approaches [27,29,53]. Our study demonstrated that, seven months post-treatment, AAV8.LSP.IVS2.eBT-IDS4 achieved 89 ​% wild-type IDS activity in the brains of MPS II mice, compared with only 29 ​% achieved by AAV8.LSP.IVS2.IDSco (Fig. 3e). Here, eBT-IDS4 contains a tandem melanotransferrin peptide. This melanotransferrin peptide retains the ability of the soluble form to cross the BBB and distribute broadly in brain parenchyma, localizing to neuronal and glial endosomes and lysosomes [55]. Studies have shown that melanotransferrin transcytosis may involve low-density lipoprotein receptor-related protein 1 (LRP1) [56,57]. The melanotransferrin peptide has been shown to bind LRP1 with high affinity and to undergo efficient transcytosis across murine brain endothelial cells [58]. These findings suggest that the enhanced brain uptake of eBT-IDS4 may be related to LRP1-mediated BBB transport. Furthermore, eBT-IDS4 may also enter the brain via blood-CSF transport routes, consistent with our observation of the protein in the choroid plexus (Fig. S6a). As previously reported, when high levels of enzymes are present in the bloodstream, a portion of the protein manages to cross the BBB, but the exact mechanism remains unclear [25,59,60]. Cardone et al. demonstrated that intravenous AAV8-TBG-IDS administration yielding supraphysiological (16- to 70-fold) plasma IDS levels fully corrected somatic pathology but only partially reduced brain GAG accumulation, indicating limited BBB penetration [25]. Our data extend these observations: both AAV8.LSP.IVS2.eBT-IDS4 and AAV8.LSP.IVS2.IDSco produced supraphysiological blood IDS activity (approximately 54- and 60-fold wild-type levels for eBT-IDS4 and IDSco, respectively), yet only AAV8.LSP.IVS2.eBT-IDS4 achieved substantial CNS enzyme delivery (89 ​%) (Fig. 3e). These results may support a dual mechanism whereby receptor-mediated transport synergizes with high circulating enzyme levels to increase brain enzyme biodistribution. Previous studies have shown that achieving 5–40 ​% wild-type brain IDS significantly reduces GAG accumulation and effectively prevents cognitive deficits [29,53,54]. Our findings were consistent with these studies: AAV8.LSP.IVS2.eBT-IDS4 treatment normalized CNS pathologies, whereas brain GAG accumulation and neuroinflammation were only significantly reduced in AAV8.LSP.IVS2.IDSco-treated mice, which resulted in an incomplete improvement in cognitive deficits.

Neuroinflammation, a hallmark of lysosomal storage disorders, likely results from the accumulation of undegraded substrates that cooperatively activate and perpetuate a neuroinflammatory milieu, which may exacerbate the disease itself. Astrogliosis is commonly observed in MPS disorders [27,35,[61], [62], [63]] and was evident in our study. Notably, AAV8.LSP.IVS2.eBT-IDS4 treatment normalized astrocytes and restored lysosomal compartment size in murine brains, whereas AAV8.LSP.IVS2.IDSco treatment only partially improved these abnormalities (Fig. 5, Fig. 6). The correction of astrogliosis achieved with AAV8.LSP.IVS2.eBT-IDS4 is comparable to what has been reported using direct intrathecal AAV9-IDS administration [54], highlighting the therapeutic potential of this brain-penetrant approach.

The DMP dry maze, which is widely used to assess spatial learning and memory (functions dependent on the hippocampus and cortex) in animal models of Alzheimer's disease and MPS I [31,36,37,[64], [65], [66]], was used in our study. AAV8.LSP.IVS2.eBT-IDS4 treatment normalized cognitive deficits, whereas AAV8.LSP.IVS2.IDSco resulted in significant, yet incomplete, improvements (Fig. 7). We hypothesized that the observed cognitive correction in AAV8.LSP.IVS2.eBT-IDS4-treated mice may stem from a combination of factors: (1) normalization of GAG storage in the brain (particularly in the hippocampus and cortex) and (2) complete elimination of astrogliosis. Importantly, this further highlights that brain-penetrant engineering of the IDS sequence is required to achieve complete correction of neurocognitive aspects in MPS II mice.

Skeletal abnormalities, among the most challenging manifestations of MPS II, likely arise from poor enzyme penetration into musculoskeletal tissues. Consistent with clinical observations, MPS II mouse model commonly exhibits progressive skeletal abnormalities, including zygomatic bone and femoral enlargement, reflecting the multiple bone malformations associated with the disease [25,38]. Previous therapeutic approaches utilizing AAV-mediated gene therapy [24,25] or hematopoietic stem cell-mediated lentiviral gene therapy [27,67,68] have achieved correction of the zygomatic arch and femoral widths in MPS II mice. In addition, hematopoietic stem and progenitor cell-mediated lentiviral gene therapy significantly improved brain and heart pathology; however, it failed to correct cartilage [69]. In our study, AAV8.LSP.IVS2.eBT-IDS4 and AAV8.LSP.IVS2.IDSco improved growth plate cartilage pathology, with complete restoration of the zygomatic arch and femoral widths to wild-type levels (Fig. 8a–c). Cardiopulmonary complications, the primary cause of death in MPS II patients [70,71], have received relatively little attention in treatment efficacy studies. Here, 8-month-old MPS II model mice exhibited significant cardiac functional defects and abnormal dilation of the ascending aorta, both of which were corrected in all treated mice (Fig. 8d–h). These findings suggest that our liver-directed AAV gene therapy enables sufficient enzyme penetration into skeletal and cardiac muscle, resulting in significant therapeutic benefits.

In summary, this study demonstrates that liver-directed AAV8.LSP.IVS2.eBT-IDS4 gene therapy effectively corrects both systemic and CNS manifestations of MPS II. These findings support the development of a minimally invasive strategy for MPS II and potentially other neurodegenerative LSDs that require widespread and sustained enzyme distribution.

## Data availability

All other data supporting the findings of this study are included in the article and its supplemental material or are available from the corresponding authors upon reasonable request.

## Author contributions

Y.Y. conceived this study; Y.Y. and X.J. designed the experiments; X.Y.W. constructed the plasmid vectors; J.S. produced the AAV8 vector; L.S. and J.M.F. performed the animal injections; and Q.X.X. and M.L. performed the ELISA. X.J., Q.Y. and X.Y.W. performed the DMP dry maze assay and micro-CT; Q.Y., M.H. and M.L. performed the echocardiography; X.J. and Q.Y. performed the immunofluorescence assay. Y.F.A., Q.Q.L. and M.J.L. performed the IDS enzyme activity assay and GAG assay; C.D.R. and F.F.L. performed the histopathology assay; X.J. wrote the manuscript; Y.Y. edited the manuscript. All the authors read and approved the final manuscript.

## Declaration of competing interest

The authors declare that they have no known competing financial interests or personal relationships that could have appeared to influence the work reported in this paper.
