# Supplementary material for: Rescue of neurologic disease in mucopolysaccharidosis type II mice via AAV-mediated liver delivery of brain-penetrating iduronate-2-sulfatase
Source: Neurotherapeutics. 2025 Sep 18;22(6):e00741. doi: 10.1016/j.neurot.2025.e00741 (PMC12664501; doi:10.1016/j.neurot.2025.e00741)
Supplement: Multimedia component 1 [file mmc1.docx]

**Supplemental Figures**

**
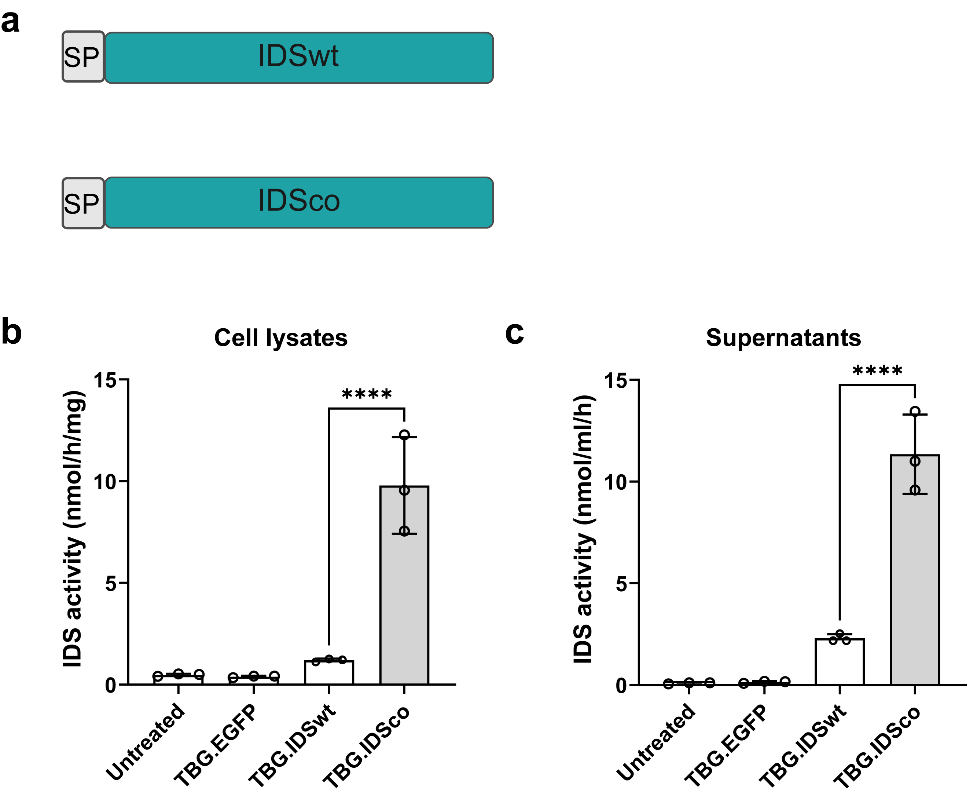
**

**Fig. S1. Codon optimization of the human IDS sequence and validation in vitro.** (a) Schematic of wild-type IDS (IDSwt) or codon-optimized IDS (IDSco) genes. SP, signal peptide. (b and c) IDS enzyme activity in HuH-7 (b) cell lysates and (c) culture supernatants after transfection. The untreated group and the plasmid-expressing EGFP-transfected group served as controls. Bars represent mean ± SD, n = 3. Significance was validated with one-way ANOVA, ****p < 0.0001 vs. TBG.IDSwt.

**
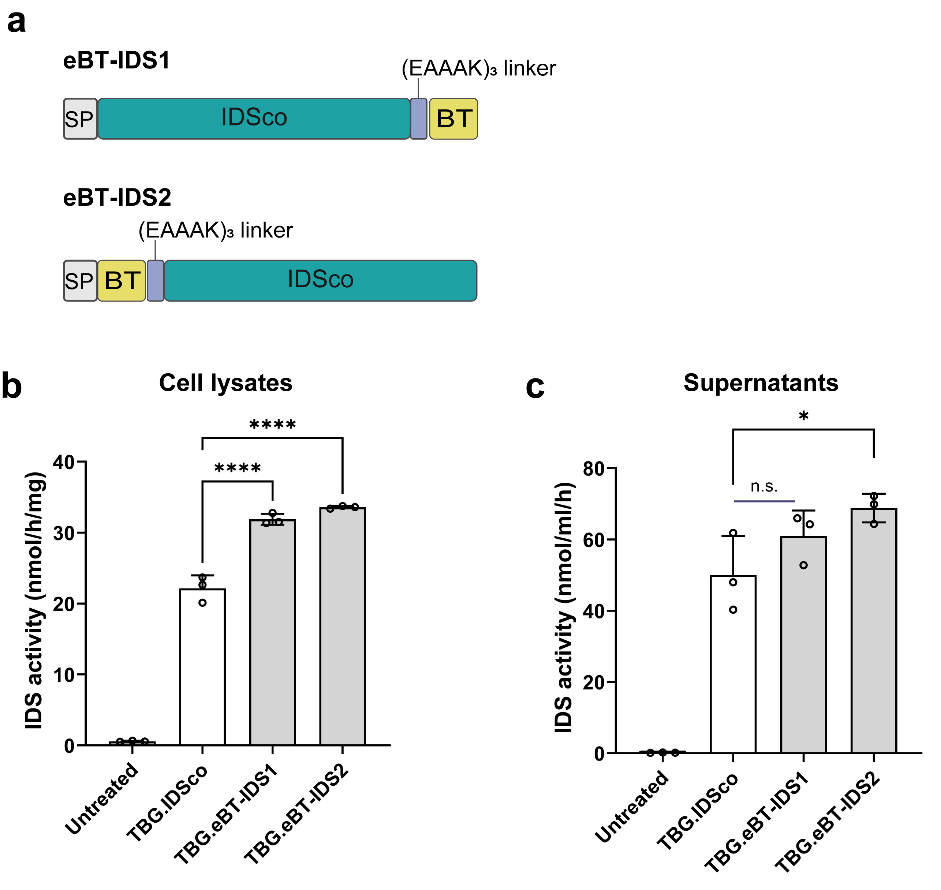
**

**Fig. S2. Engineering of brain-penetrant IDS and validation in vitro.** (a) Schematic of the eBT-IDS1 and eBT-IDS2 genes. SP, signal peptide; IDSco, codon-optimized IDS; BT, brain-targeted peptide (melanotransferrin peptide). (b and c) IDS enzyme activity in HuH-7 (b) cell lysates and (c) culture supernatants after transfection. The untreated group served as a control. Bars represent mean ± SD, n = 3. Significance was validated with one-way ANOVA, n.s. = not significant, *p<0.05, ****p<0.0001 vs. TBG.IDSco.

**
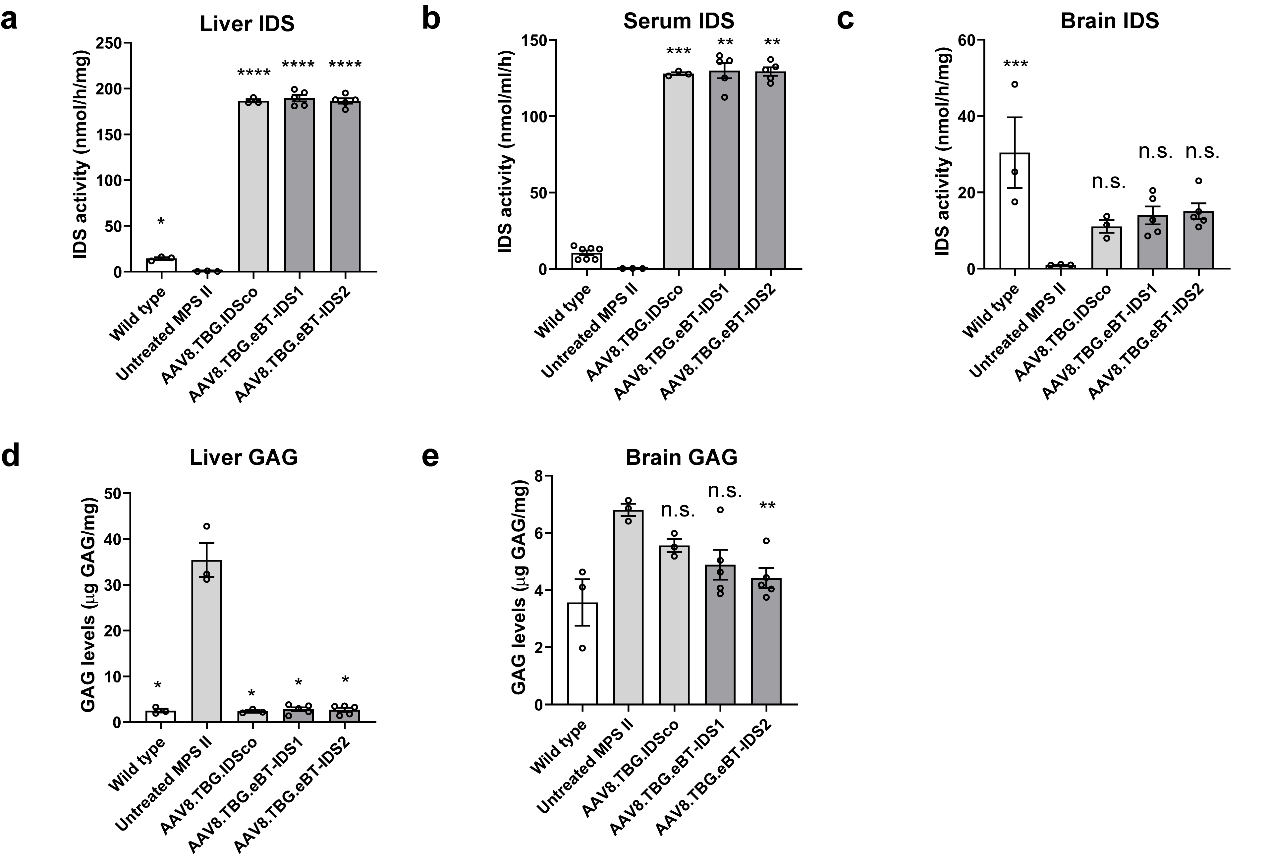
**

**Fig. S3. Engineering of brain-penetrant IDS and validation in vivo.** (a-c) IDS enzyme activity in the (a) liver, (b) serum and (c) brain at 2 months post-injection. (d and e) GAG levels in the (d) liver and (e) brain at 2 months post-injection. Wild-type and untreated mice served as controls. Bars represent mean ± SEM, n = 3-7 mice/group. Significance was validated with one-way ANOVA, n.s. = not significant; *p<0.05, **p<0.01, ***p<0.001, ****p<0.0001 vs. untreated MPS II.

**
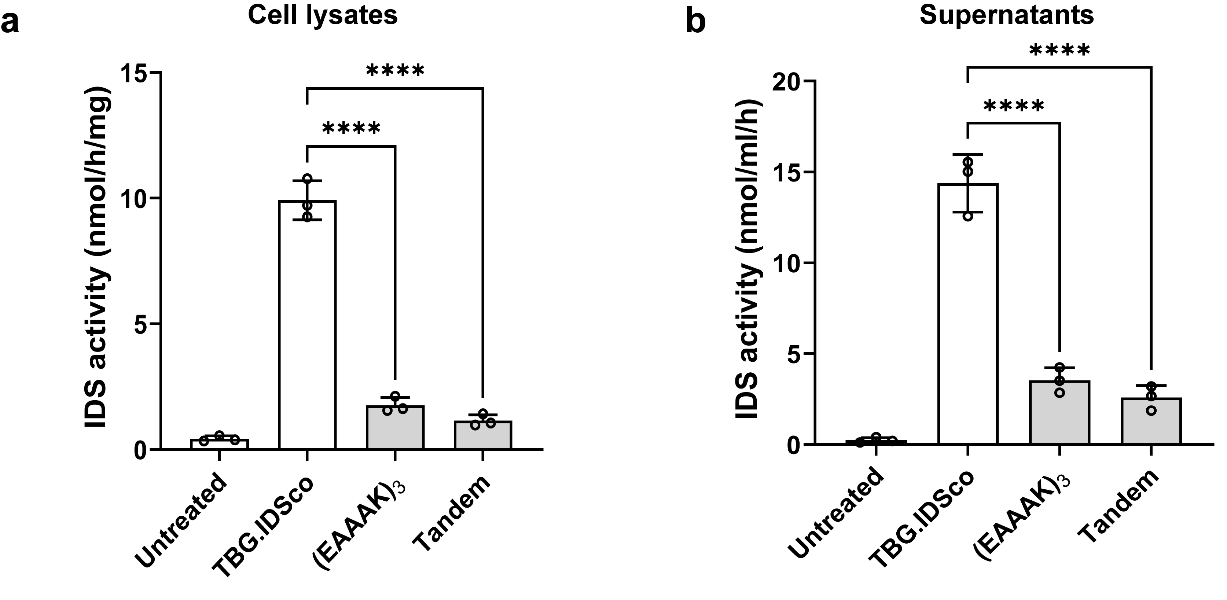
**

**Fig. S4. Fusion of two melanotransferrin peptides and validation in vivo.** (a and b) IDS enzyme activity in HuH-7 (a) cell lysates and (b) culture supernatants after transfection. In vitro validation of two melanotransferrin peptides linked by direct tandem or an (EAAAK)_3_ rigid linker. The untreated group served as a control. Bars represent mean ± SD, n = 3. Significance was validated with one-way ANOVA, ****p<0.0001 vs. TBG.IDSco.


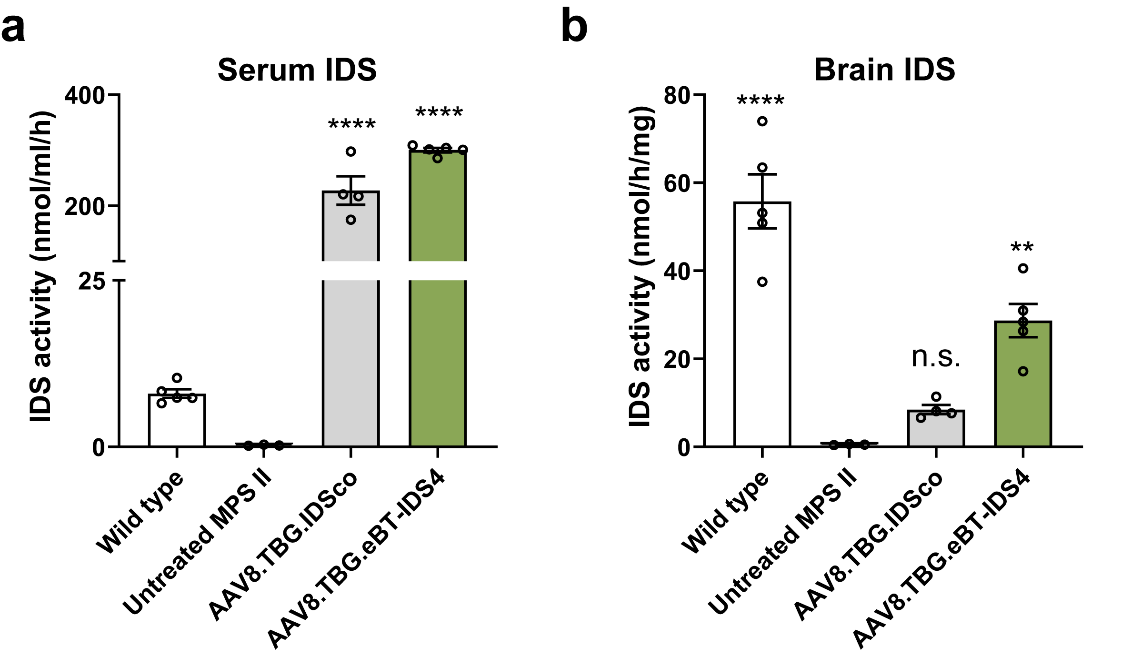


**Fig. S5. AAV8.TBG.eBT-IDS4 significantly restores IDS enzyme activity in brain tissues at two weeks post-treatment.** (a and b) IDS enzyme activity in the (a) serum and (b) brain at two weeks post-treatment. Wild-type and untreated mice served as controls, n = 3-5 mice/group. Bars represent mean ± SEM. Significance was validated with one-way ANOVA, n.s. = not significant, **p<0.01, ****p<0.0001 vs. untreated MPS II.

**
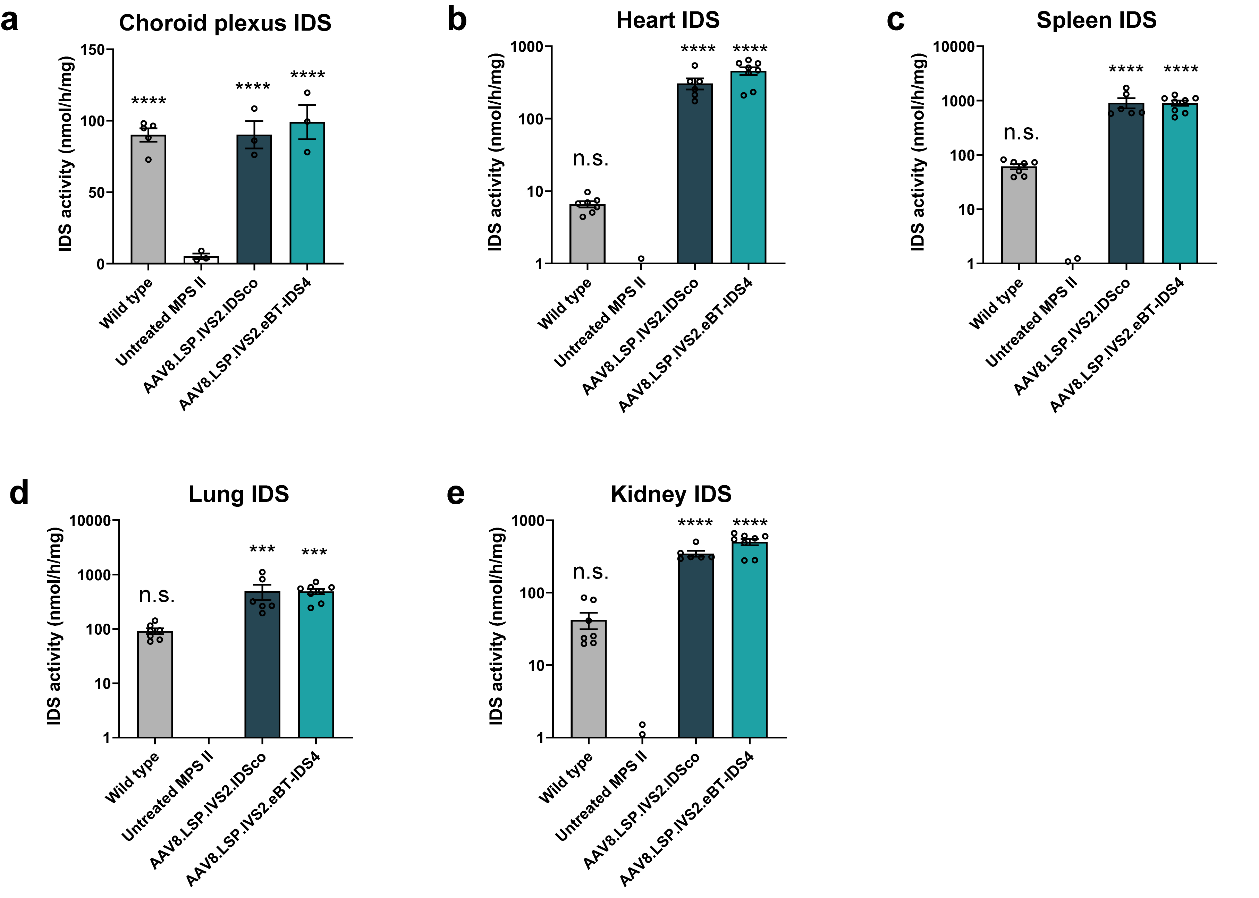
**

**Fig. S6. AAV8.LSP.IVS2.eBT-IDS4 and AAV8.LSP.IVS2.IDSco significantly restores IDS enzyme activity in the choroid plexus and peripheral tissues.** (a) IDS enzyme activity in the choroid plexus at 8 months post-treatment. Wild-type and untreated mice served as controls, n = 3-5 mice/group. (b-e) IDS enzyme activity in tissues at 7 months post-treatment, including (b) heart, (c) spleen, (d) lung, and (e) kidney. Wild-type and untreated mice served as controls, n = 6-8 mice/group. (a-e) Bars represent mean ± SEM. Significance was validated with one-way ANOVA, n.s. = not significant, ***p<0.001, ****p<0.0001 vs. untreated MPS II.

**
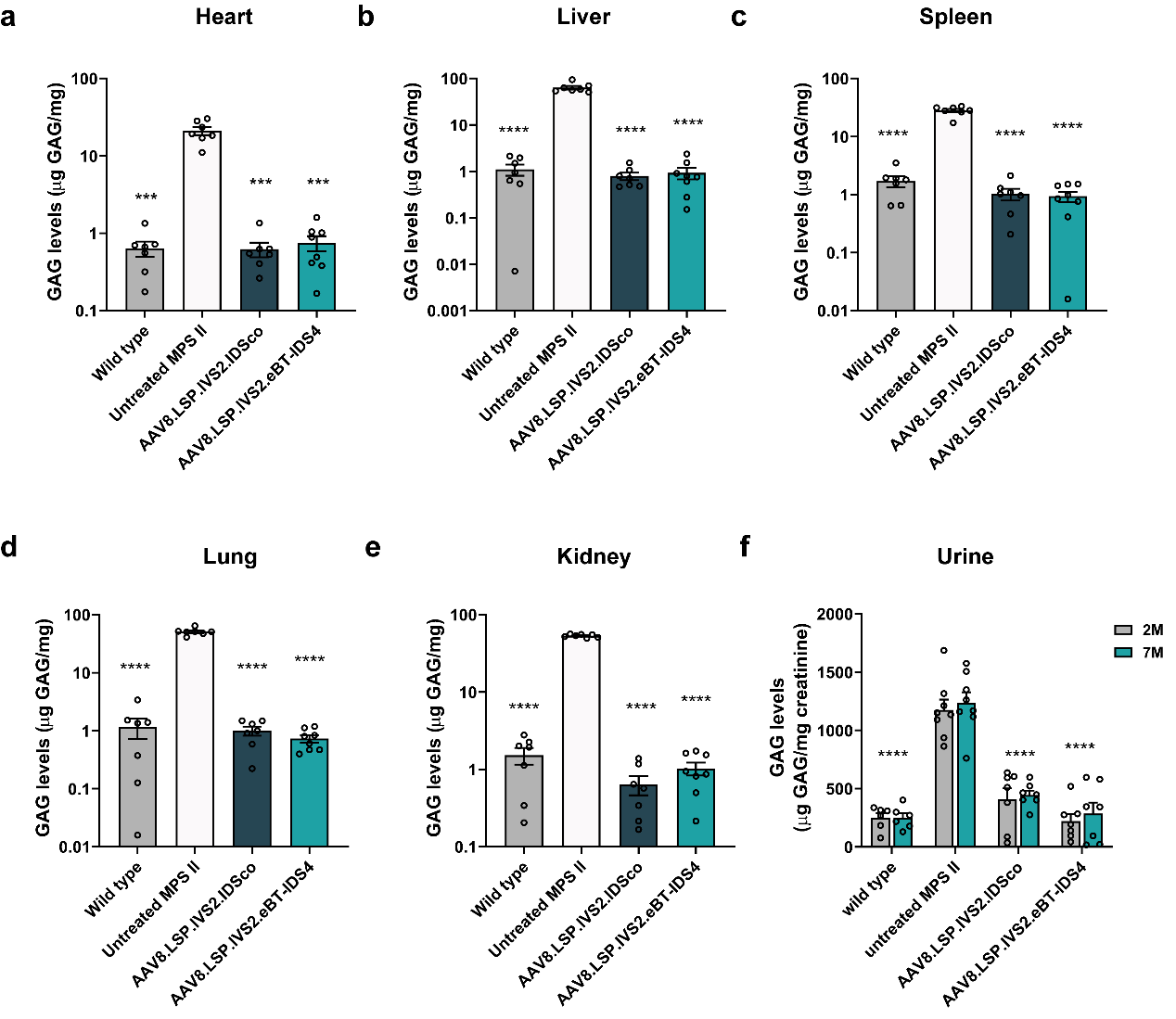
**

**Fig. S7. AAV8.LSP.IVS2.eBT-IDS4 and AAV8.LSP.IVS2.IDSco normalizes GAG levels in peripheral tissues.** (a-f) GAG levels in the (a) heart, (b) liver, (c) spleen, (d) lung, (e) kidney and (f) urine at 7 months post-treatment. Wild-type and untreated mice served as controls. Bars represent mean ± SEM, n = 6-8 mice/group. Significance was validated with one-way ANOVA, ***p<0.001, ****p<0.0001 vs. untreated MPS II.

**
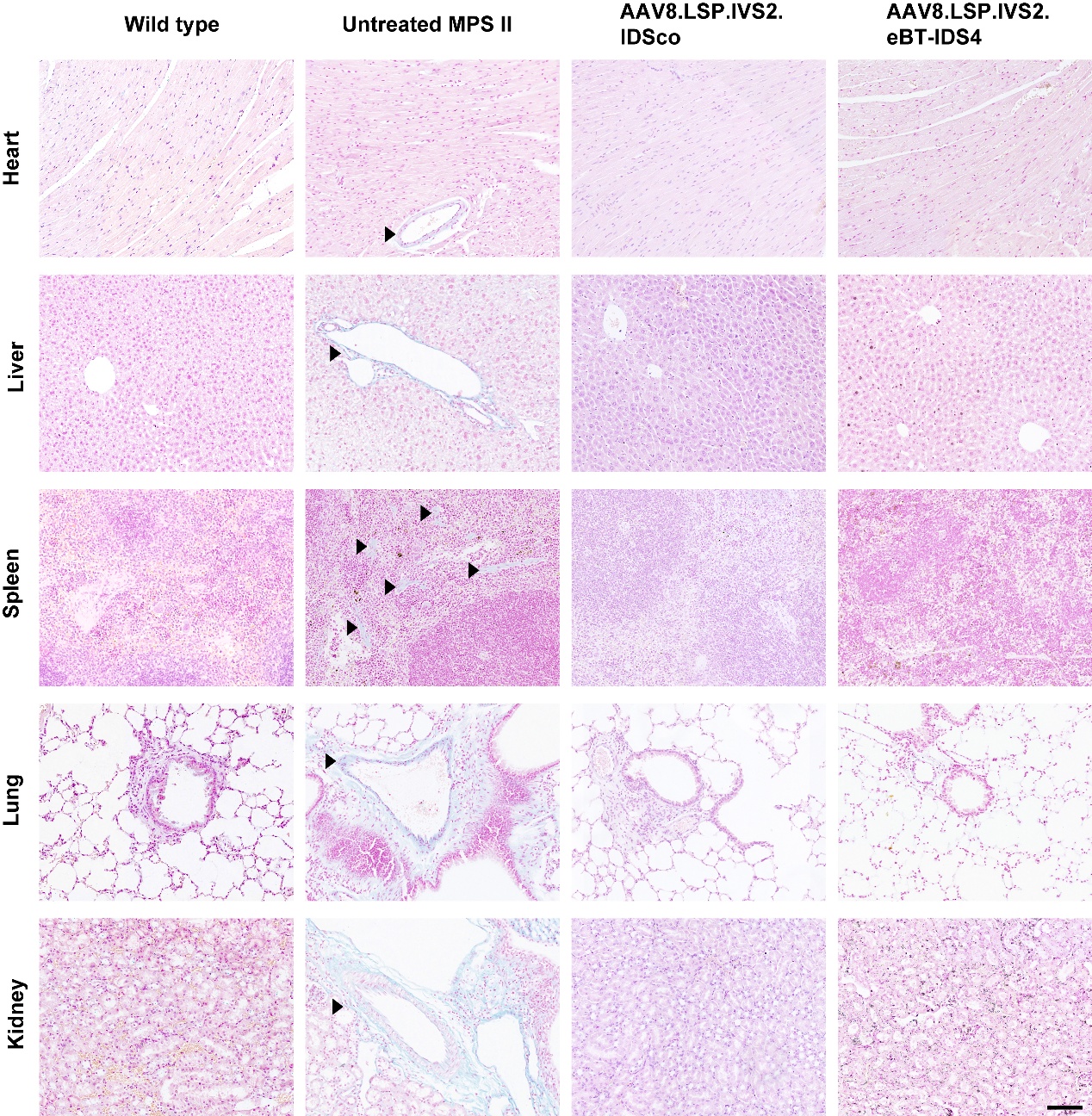
**

**Fig. S8. AAV8.LSP.IVS2.eBT-IDS4 and AAV8.LSP.IVS2.IDSco corrects GAG pathology in peripheral tissues.** Alcian blue staining of the heart, liver, spleen, lung and kidney at 7 months post-treatment. Scale bars: 100 μm. Black triangles indicate GAGs stained blue.

**
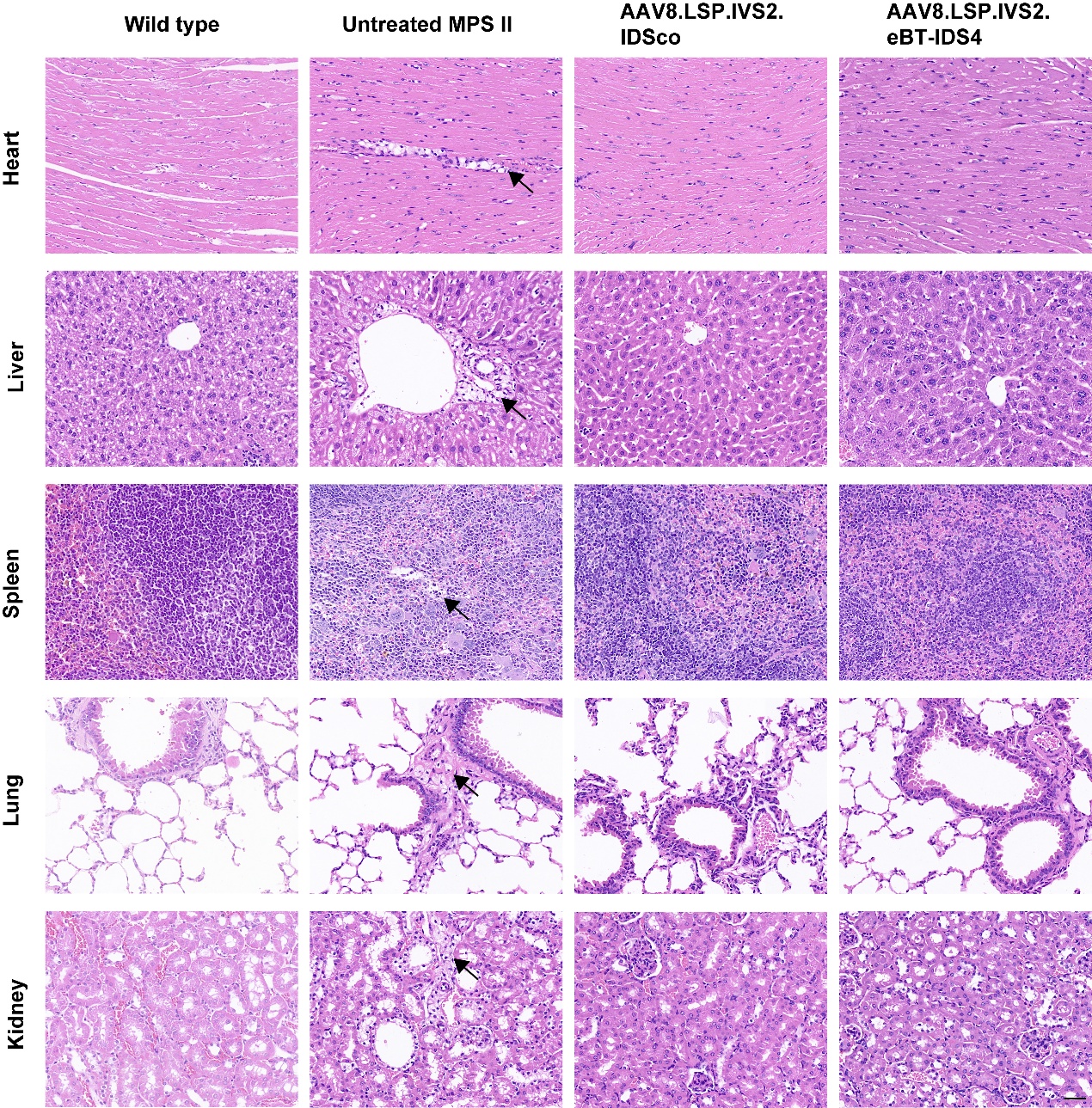
**

**Fig. S9. AAV8.LSP.IVS2.eBT-IDS4 and AAV8.LSP.IVS2.IDSco corrects histological abnormalities in peripheral tissue.** H&E staining of the heart, liver, spleen, lung and kidney at 7 months post-treatment. Scale bars: 40 μm. Black arrows indicate foamy macrophages.

**
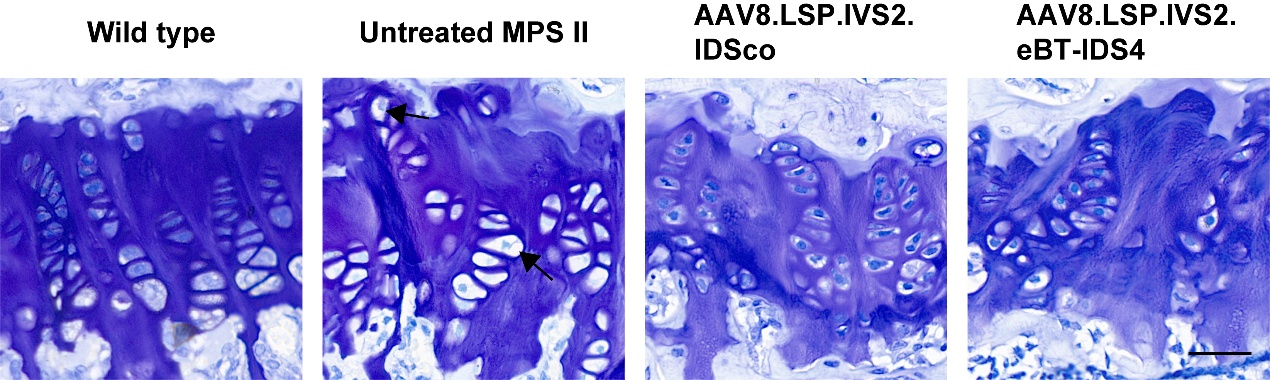
**

**Fig. S10. AAV8.LSP.IVS2.eBT-IDS4 and AAV8.LSP.IVS2.IDSco improves cartilage pathology.** Toluidine blue staining shows chondrocyte vacuolization in the femoral growth plate of 8-month-old MPS II mice (black arrows). Scale bars: 40 μm.

**
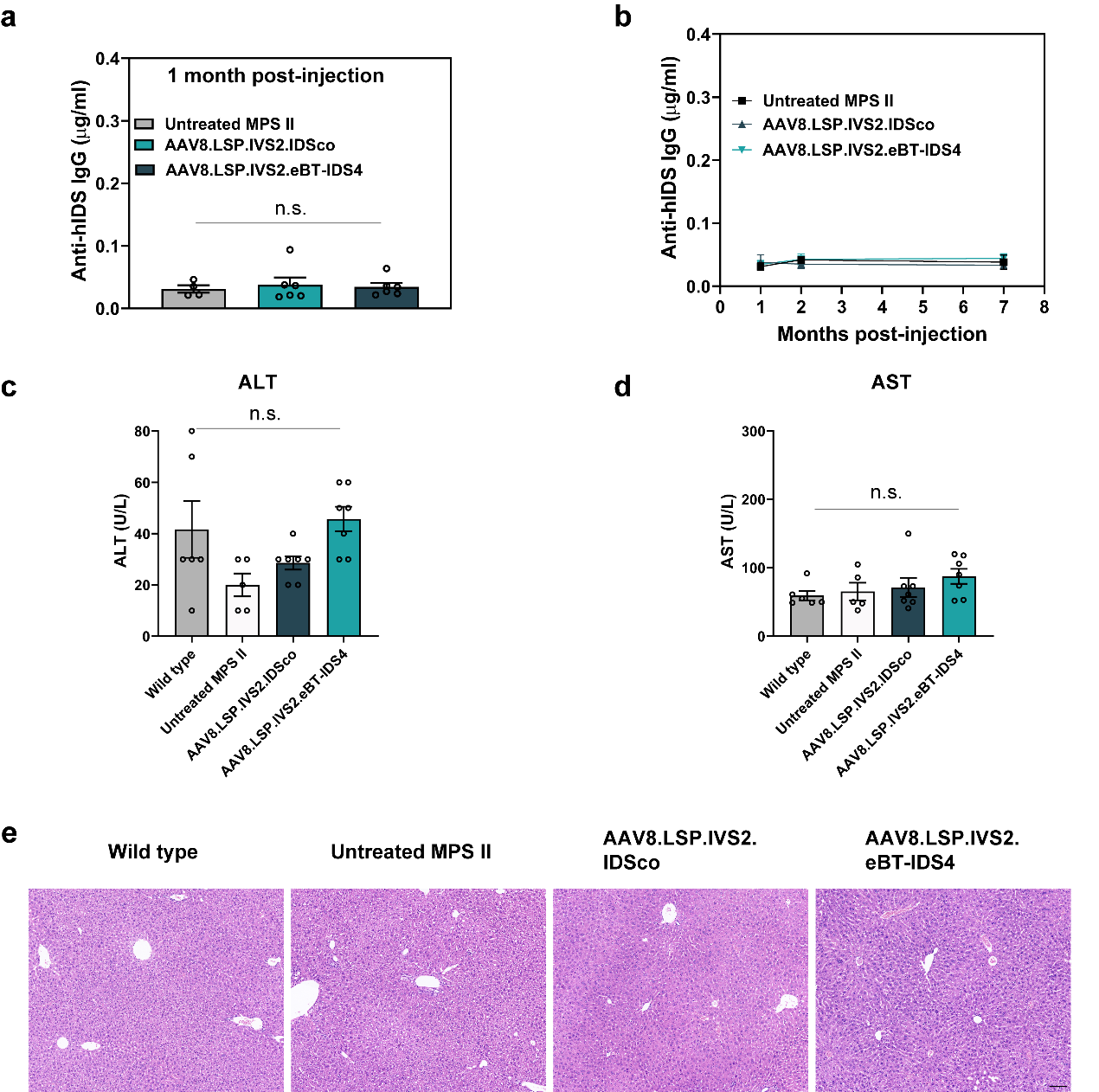
s**

**Fig. S11. Hepatotoxicity and human IDS antibody analyses in AAV8.LSP.IVS2.eBT-IDS4- and AAV8.LSP.IVS2.IDSco-treated mice.** (a and b) Human IDS IgG antibody titers in serum at (a) one month post-treatment and (b) throughout the treatment period. Bars represent mean ± SEM, n = 4-6 mice/group. Significance was validated with one-way ANOVA, n.s. = not significant vs. untreated MPS II. (c and d) Serum ALT and AST levels at 7 months post-treatment. Bars represent mean ± SEM, n = 5-7 mice/group. Significance was validated with one-way ANOVA, n.s. = not significant vs. wild type. (e) H&E staining of liver sections at 7 months post-treatment. Scale bars: 100 μm.

**Supplemental Tables**

**Table S1. The nucleotide sequences of peptide and linkers.**

| **Name** | **sequence** |
| --- | --- |
| Melanotransferrin peptide | gacagcagccatgcctttaccctggacgagctgaga |
| (EAAAK)_3_ | GAGGCCGCTGCTAAAGAGGCTGCCGCCAAAGAAGCCGCCGCTAAG |
| XTEN | TCCGGCAGCGAGACGCCAGGCACCTCCGAGAGCGCTACGCCTGAATCC |

**Supplemental Sequences**

Signal peptide is orange, melanotransferrin peptide is pink, (EAAAK)_3_ linker is blue and XTEN linker is green.

**Amino acid sequences of eBT-IDS1**

MPPPRTGRGLLWLGLVLSSVCVALGSETQANSTTDALNVLLIIVDDLRPSLGCYGDKLVRSPNIDQLASHSLLFQNAFAQQAVCAPSRVSFLTGRRPDTTRLYDFNSYWRVHAGNFSTIPQYFKENGYVTMSVGKVFHPGISSNHTDDSPYSWSFPPYHPSSEKYENTKTCRGPDGELHANLLCPVDVLDVPEGTLPDKQSTEQAIQLLEKMKTSASPFFLAVGYHKPHIPFRYPKEFQKLYPLENITLAPDPEVPDGLPPVAYNPWMDIRQREDVQALNISVPYGPIPVDFQRKIRQSYFASVSYLDTQVGRLLSALDDLQLANSTIIAFTSDHGWALGEHGEWAKYSNFDVATHVPLIFYVPGRTASLPEAGEKLFPYLDPFDSASQLMEPGRQSMDLVELVSLFPTLAGLAGLQVPPRCPVPSFHVELCREGKNLLKHFRFRDLEEDPYLPGNPRELIAYSQYPRPSDIPQWNSDKPSLKDIKIMGYSIRTIDYRYTVWVGFNPDEFLANFSDIHAGELYFVDSDPLQDHNMYNDSQGGDLFQLLMPEAAAKEAAAKEAAAKDSSHAFTLDELR*

**Amino acid sequences of eBT-IDS2**

MPPPRTGRGLLWLGLVLSSVCVALGDSSHAFTLDELREAAAKEAAAKEAAAKSETQANSTTDALNVLLIIVDDLRPSLGCYGDKLVRSPNIDQLASHSLLFQNAFAQQAVCAPSRVSFLTGRRPDTTRLYDFNSYWRVHAGNFSTIPQYFKENGYVTMSVGKVFHPGISSNHTDDSPYSWSFPPYHPSSEKYENTKTCRGPDGELHANLLCPVDVLDVPEGTLPDKQSTEQAIQLLEKMKTSASPFFLAVGYHKPHIPFRYPKEFQKLYPLENITLAPDPEVPDGLPPVAYNPWMDIRQREDVQALNISVPYGPIPVDFQRKIRQSYFASVSYLDTQVGRLLSALDDLQLANSTIIAFTSDHGWALGEHGEWAKYSNFDVATHVPLIFYVPGRTASLPEAGEKLFPYLDPFDSASQLMEPGRQSMDLVELVSLFPTLAGLAGLQVPPRCPVPSFHVELCREGKNLLKHFRFRDLEEDPYLPGNPRELIAYSQYPRPSDIPQWNSDKPSLKDIKIMGYSIRTIDYRYTVWVGFNPDEFLANFSDIHAGELYFVDSDPLQDHNMYNDSQGGDLFQLLMP*

**Amino acid sequences of eBT-IDS3**

MPPPRTGRGLLWLGLVLSSVCVALGSETQANSTTDALNVLLIIVDDLRPSLGCYGDKLVRSPNIDQLASHSLLFQNAFAQQAVCAPSRVSFLTGRRPDTTRLYDFNSYWRVHAGNFSTIPQYFKENGYVTMSVGKVFHPGISSNHTDDSPYSWSFPPYHPSSEKYENTKTCRGPDGELHANLLCPVDVLDVPEGTLPDKQSTEQAIQLLEKMKTSASPFFLAVGYHKPHIPFRYPKEFQKLYPLENITLAPDPEVPDGLPPVAYNPWMDIRQREDVQALNISVPYGPIPVDFQRKIRQSYFASVSYLDTQVGRLLSALDDLQLANSTIIAFTSDHGWALGEHGEWAKYSNFDVATHVPLIFYVPGRTASLPEAGEKLFPYLDPFDSASQLMEPGRQSMDLVELVSLFPTLAGLAGLQVPPRCPVPSFHVELCREGKNLLKHFRFRDLEEDPYLPGNPRELIAYSQYPRPSDIPQWNSDKPSLKDIKIMGYSIRTIDYRYTVWVGFNPDEFLANFSDIHAGELYFVDSDPLQDHNMYNDSQGGDLFQLLMPEAAAKEAAAKEAAAKDSSHAFTLDELRSGSETPGTSESATPESDSSHAFTLDELR*

**Amino acid sequences of eBT-IDS4**

MPPPRTGRGLLWLGLVLSSVCVALGDSSHAFTLDELRSGSETPGTSESATPESDSSHAFTLDELREAAAKEAAAKEAAAKSETQANSTTDALNVLLIIVDDLRPSLGCYGDKLVRSPNIDQLASHSLLFQNAFAQQAVCAPSRVSFLTGRRPDTTRLYDFNSYWRVHAGNFSTIPQYFKENGYVTMSVGKVFHPGISSNHTDDSPYSWSFPPYHPSSEKYENTKTCRGPDGELHANLLCPVDVLDVPEGTLPDKQSTEQAIQLLEKMKTSASPFFLAVGYHKPHIPFRYPKEFQKLYPLENITLAPDPEVPDGLPPVAYNPWMDIRQREDVQALNISVPYGPIPVDFQRKIRQSYFASVSYLDTQVGRLLSALDDLQLANSTIIAFTSDHGWALGEHGEWAKYSNFDVATHVPLIFYVPGRTASLPEAGEKLFPYLDPFDSASQLMEPGRQSMDLVELVSLFPTLAGLAGLQVPPRCPVPSFHVELCREGKNLLKHFRFRDLEEDPYLPGNPRELIAYSQYPRPSDIPQWNSDKPSLKDIKIMGYSIRTIDYRYTVWVGFNPDEFLANFSDIHAGELYFVDSDPLQDHNMYNDSQGGDLFQLLMP*
